# The neotypification of *Frontonia vernalis* (Ehrenberg, 1833) Ehrenberg, 1838 and the description of *Frontonia paravernalis* sp. nov. trigger a critical revision of frontoniid systematics

**DOI:** 10.1186/s40850-021-00067-9

**Published:** 2021-03-08

**Authors:** Valentina Serra, Aldo D’Alessandro, Venkatamahesh Nitla, Leandro Gammuto, Letizia Modeo, Giulio Petroni, Sergei I. Fokin

**Affiliations:** 1 Department of Biology, University of Pisa, Pisa, Italy; 2 School of Biosciences and Veterinary Medicine, University of Camerino, Camerino, Italy; 3 CIME, Centro Interdipartimentale di Microscopia Elettronica, Università di Pisa, Pisa, Italy; 4 CISUP, Centro per l’Integrazione della Strumentazione dell’Università di Pisa, Pisa, Italy; 5 Department of Invertebrate Zoology, St. Petersburg State University, St. Petersburg, Russia; 6 St. Petersburg Branch of the S.I. Vavilov Institute of History of Science and Technology Russian Academy of Sciences, St. Petersburg, Russia

**Keywords:** Peniculia, Phylogeny, Green frontoniids, *Frontonia vernalis*, *Frontonia leucas*, Neotypification, Taxonomy

## Abstract

**Background:**

Among Oligohymenophorea (Ciliophora, Alveolata) the subclass Peniculia stands as one of the most well-known groups. *Frontonia* is the largest genus of Peniculia, and its representatives are spread in any type of water bodies as well as in soil. At a first glance, *Frontonia* species exhibit an overall similar morphology, and form a well-recognizable taxon of ciliates. Despite the general morphological homogeneity, the phylogenetic analysis based on the 18S rDNA sequencing showed that *Frontonia* is a non-monophyletic group. The systematics of this genus should be deeply reviewed, although additional issues complicate the task solving. First, type species of the genus is not yet clearly established, and no type material is available. In this context, the situation of *F. vernalis*, one of the first *Frontonia* ever described, is somehow puzzled: the description of this ciliate made by Ehrenberg (in 1833 and 1838) contains several inaccuracies and subsequent misidentifications by other authors occurred. Moreover, the 18S rDNA sequence of a putative *F. vernalis* is available on GenBank, but no morphological description of the correspondent specimens is provided; thus, in our opinion, it should be only prudently associated with *F. vernalis* or at least indicated as “*F. vernalis*”.

**Results:**

In the present work, we provide the neotypification of *F. vernalis* newly found in Italy, presenting its multidisciplinary description and its neotype material. Similarly, we describe a novel species bearing *Chlorella*-like endosymbionts, *Frontonia paravernalis* sp. nov., retrieved in two far distant locations (Italy, Russia). A critical discussion on the status of *Frontonia* taxonomy and phylogeny is also presented, based on the 18S rDNA sequencing of both these two newly collected species and other 14 frontoniids isolated in different parts of the world. Finally, in the present study *F. leucas* was neotypified and proposed as the type species of the genus.

**Conclusions:**

Green frontoniids form a monophyletic clade of freshwater organisms characterized by having a single contractile vacuole and bearing intracytoplasmatic *Chlorella*-like symbionts. With the neotypification of *F. vernalis* and *F. leucas* a fundamental step in *Frontonia* systematics was taken, and the bases for further taxonomic studies were laid.

**Supplementary Information:**

The online version contains supplementary material available at 10.1186/s40850-021-00067-9.

## Background

Among Oligohymenophorea (Ciliophora, Alveolata) the subclass Peniculia is one of the most well-known and, in some respects, actively studied group of ciliates. Some peniculines, such as members of the *Paramecium* genus, serve as useful model organisms in protistology and other biological disciplines since time. Moreover, Peniculia is one of the most common group of ciliates, retrieved in many and different biotopes, in particular highly represented by *Frontonia* and *Paramecium* [1–17]. *Frontonia* is the largest genus of Peniculia, comprising, according to literature data, more than 40 species. However, only some of them are relatively common [3, 7, 11, 15, 16, 18–25]. *Frontonia* representatives are widely spread in any type of water bodies (freshwater, brackish water and marine) as well as in soil [7].

At a first glance, *Frontonia* members exhibit an overall similar morphology, forming a well-recognizable group of ciliates. Shared characters are: i) a more anterior location of cytostome with respect to its position in *Paramecium*; ii) the bucco-kinetal type of stomatogenesis, with a distinctive set of oral membranelles (so-called “oligohymenium”) consisting in three peniculi on the left side of the buccal cavity plus a paroral membrane on the right buccal margin; iii) a somatic ciliature uniformly organized (except for some vestibular and postoral kineties) with distinctive pre- and postoral sutures; and iv) a typical kind of extrusome (trichocyst, according to their similarity with trichocyts of *Paramecium*) inserted in the cell cortex. At the same time, other morphological features appear strongly variable within the group (e.g., number and structure of contractile vacuoles, composition of buccal ciliature, micronuclei number and type, and cell size/shape). Despite the general morphological homogeneity, the phylogenetic analysis based on 18S rDNA sequences showed that *Frontonia* is a non-monophyletic group [26, 27]. This result has been confirmed using different sets of frontoniids and by different research groups (e.g., [11, 15, 16, 28]). Thus, it can be hypothesised that the “frontoniid” morphotype could be the result of a sum of plesiomorphies retained in different lineages of peniculines. This situation caused the present paraphyly of the genus.

**Fig. 1:**
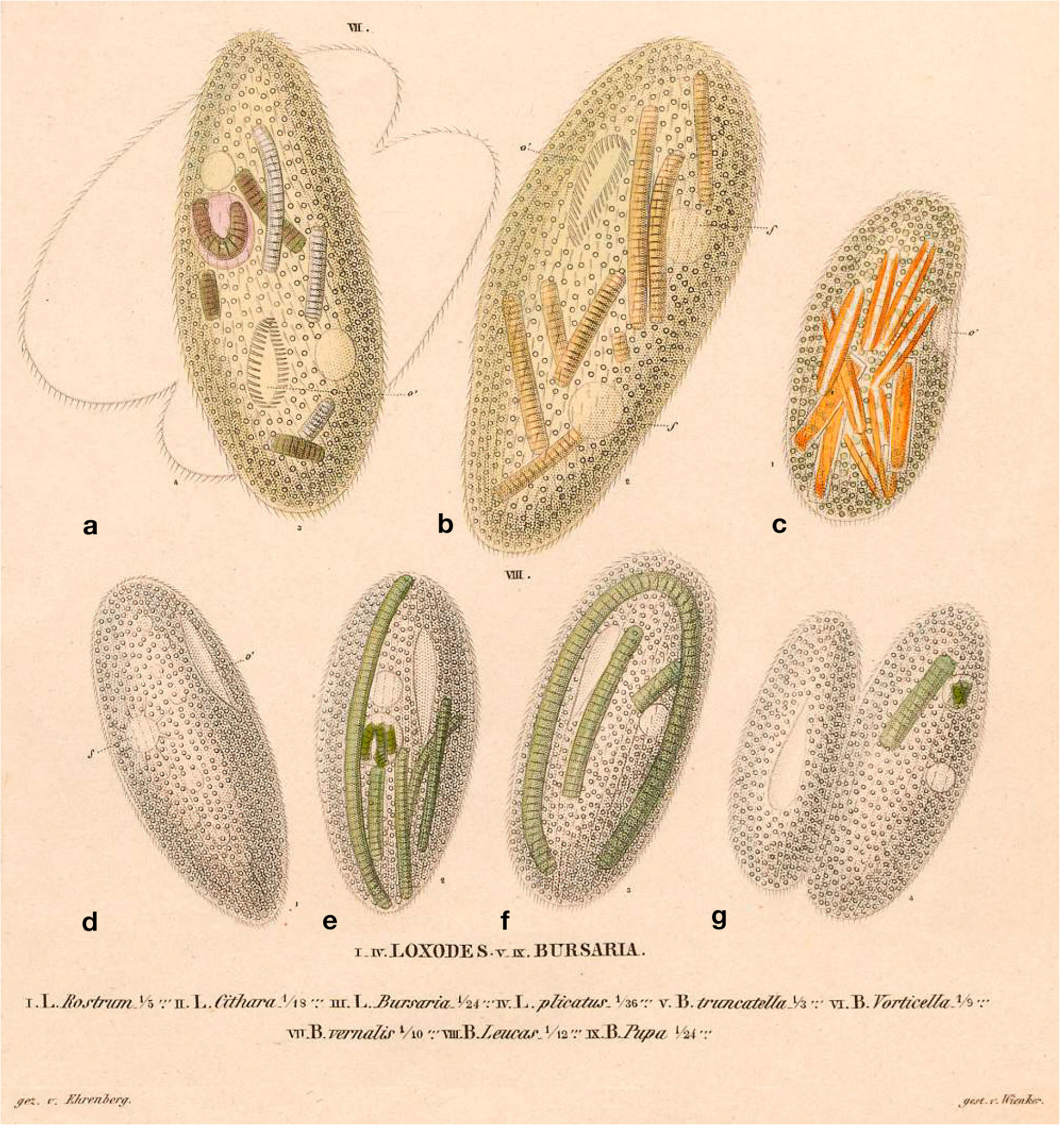
Original drawing of *Frontonia vernalis* (**a**-**c**) and *F. leucas* (**d**-**g**) made by Ehrenberg in 1838: part of Plate 34, Figs VII, VIII (Ehrenberg, 1838 - Atlas). In this study Ehrenberg simply reproduced the main part of drawing of *F. vernalis* made by himself in 1833 ([29]: p. 383, Plate III). **a**, **b** Two large green cells in which two vacuoles are depicted, but without collecting canals. Behind the first *F. vernalis* cell (**a**), there is the profile of a conjugating pair, which the Author interpreted as a dividing cell; **c** a much smaller green cell, in which only a single contractile vacuole is visible; **d**, **e**, **f** three white cells of *F. leucas* with one contractile vacuole each. In the left most cell (**d**) collecting canals of contractile vacuole are visible; **g** conjugating pair of *F. leucas*, which again was interpreted by the Author as a dividing cell. Small transparent drops spread over the cells of *F. vernali*s and *F. leucas* are, apparently, trichocysts. The image is not copyrighted due to the age of the work

It is our opinion that the systematics of this genus should be deeply reviewed, although we realized that some issues complicate even more the task. First of all, the type species of the genus is not yet clearly established, and at present no type material is available. Indeed, *Frontonia leucas* (Ehrenberg, 1833) Ehrenberg, 1838, one of the first described *Frontonia* species which could be eligible to this role, actually consists of a set of different species, morphologically close to each other [3, 9]. In addition, also the other species described by Ehrenberg in 1833, *Frontonia vernalis* (Ehrenberg, 1833) Ehrenberg, 1838, presents a somehow puzzled situation. Actually, it was described by Ehrenberg as a freshwater species, 211–254 μm long, bearing *Chlorella*-like cytoplasmic endosymbionts, and carrying two contractile vacuoles (CVs) (Fig. 1) [29, 30]. Unfortunately, subsequent misidentifications by other authors occurred [17]: for instance, *F. vernalis* sensu Bullington [31] cannot be considered coincident with *F. vernalis* sensu Ehrenberg, because Bullington described a brackish water *Frontonia* species without *Chlorella*-like symbionts, morphologically closer to the *F. fusca* described by Quennerstedt [32] and recently redescribed by Fokin [10].

Additionally, molecular data regarding *F. vernalis* are somehow confusing as well. As for many other *Frontonia*’s 18S rDNA sequences present in on-line databases, the sequence of a putative *F. vernalis* available on GenBank (accession number U97110) is not linked to any morphological description. This sequence is the only one available for the species and was deposited by Hirt and colleagues in 1997, not supported by any publication. In fact, this ciliate isolated in England (UK) has never been morphologically described. Moreover, it is known that Hirt and colleagues worked with freshwater frontoniids hosting *Chlorella*-like cytoplasmic symbionts (e.g., [33–35]) detected in a small productive pond (Priest Pot, Like District, Cumbria, England, UK), although these organisms, according to few pictures presented in their publications, probably do not match the original morphotype of *F. vernalis* for which Ehrenberg mentioned the presence of two CVs (Fig. 1). In this context, we suggest that the sequence U97110 should not be reliably associated with *F. vernalis,* since a description based on a multidisciplinary study approach [16, 36, 37] of the corresponding organism is lacking.

It is worth noting that, to date, nobody has succeeded to find a ciliate corresponding to the Ehrenberg’s *F. vernalis* (i.e., showing two CVs), although “green” *Frontonia* ciliates with a single CV were repeatedly reported in the literature [4, 15, 18, 22, 33, 38–41].

In the present work, we propose the neotypification of *F. vernalis*, deeply analysing the available literature on *Frontonia* species bearing intracytoplasmic green algae (from now on “green *Frontonia* spp*.*” or “green frontoniids”), with a careful revision and re-interpretation of data presented by Ehrenberg. Along with *F. vernalis*, we found and described by means of a multimethod study [16, 36, 37] a novel species of green frontoniid, *F. paravernalis* sp. nov., closely related to the *F. vernalis* group. Moreover, based on the 18S rDNA sequences of the endosymbionts of *F. vernalis* and *F. paravernalis*, a reconstruction of the phylogenetic relationships within the *Chlorella*-clade is provided.

A critical discussion about the current status of the *Frontonia* taxonomy and phylogeny is presented as well. To better accomplish this goal we included in the analysis the 18S rDNA sequences we produced in the last 15 years from other 14 frontoniids isolated in different parts of the world (Table 1), with a corresponding morphological diagnosis of each retrieved species. Among them, at least two resulted new species, and four of them were already known frontoniids (i.e., *F. atra, F. fusca*, *F. minuta*, and *F. vesiculosa*) for which the gene sequence was not yet published.

**Table 1:** Sampling information and accession numbers of *Frontonia* species from the present study

Species	Population	Sampling site	Country	Coordinates	Year	Habitat	Seq. ID
*Frontonia vernalis***- Neotype**	IPSal+b	Mouth of Serchio River, Pisa	Italy	N. 43° 47′ 7.524″ E. 10° 15′ 57.44”	2017	Freshwater	MT040840
*Frontonia* sp.^a^	FSPBb	Old Peterhof, St. Petersburg	Russia	N. 59° 52′ 45.88′′ E. 29° 51′ 37.224′′	2016	Freshwater	NO DATA
*Frontonia paravernalis*	IPSal+sm	Mouth of Serchio River, Pisa	Italy	N. 43° 47′ 7.524″ E. 10° 15′ 57.44”	2017	Freshwater	MT040839
*Frontonia paravernalis*	FSPBsm	Old Peterhof, St. Petersburg	Russia	N. 59° 52′ 45.88′′ E. 29° 51′ 37.224′′	2016	Freshwater	MT040838
*Frontonia* sp.	IPSal-	Mouth of Serchio River, Pisa	Italy	N. 43° 47′ 7.524″ E. 10° 15′ 57.44”	2017	Freshwater	MT040841
*Frontonia* sp.	VmFr	Monte Urpino Park, Cagliari	Italy	N. 39° 13′ 2.716″ E. 9° 8′ 1.345”	2017	Freshwater	MT040842
*Frontonia* sp.	BJ4	Balugoan Jetty, Chilka Lake, Odisha	India	N. 19° 44′ 37.021″ E. 85° 12′ 44.398”	2014	Brackish water	MT040843
*Frontoni atra*	F4	Mouth of Serchio River, Pisa	Italy	N. 43° 47′ 7.524″ E. 10° 15′ 57.44”	2005	Freshwater	MT040844
*Frontonia fusca*	F3	Mouth of Serchio River, Pisa	Italy	N. 43° 47′ 7.524″ E. 10° 15′ 57.44”	2005	Brackish water	MT040845
*Frontonia leucas*	IPBG	Pond in Botanical garden of University of Pisa, Pisa	Italy	N. 43° 43′ 10.97″ E. 10° 23′ 45.387”	2005	Freshwater	AM072622
*Frontonia leucas*	KNP3	Kolleru lake, Andhra Pradesh	India	N. 16° 43′ 10.2″ E. 81° 19′ 35.2”	2016	Freshwater	KY855558
*Frontonia minuta*	F2	Mouth of Serchio River, Pisa	Italy	N. 43° 47′ 7.524″ E. 10° 15′ 57.44”	2005	Freshwater	MT040846
*Frontonia paramagna*	KTC4	Kolleru lake, Andhra Pradesh	India	N. 16° 43′ 18.1″ E. 81° 19′ 37.3”	2014	Freshwater	KY855559
*Frontonia paramagna*	KKR19	Kolleru lake, Andhra Pradesh	India	N. 16° 43′ 10.2″ E. 81° 19′ 35.2”	2014	Freshwater	KY855554
*Frontonia paramagna*	ML	Mudasarlova Garden, Visakhapatnam, Andhra Predesh	India	N. 17° 45′ 43.175″ E. 83° 17′ 47.302”	2015	Freshwater	LT628495
*Frontonia paramagna*	BDM3	Boddam pond, Andhra Pradesh	India	N. 18° 3′ 59.425″ E. 83° 8′ 34.346”	2014	Freshwater	MT040847
*Frontonia paramagna*	KT1	Kottavuro pond, Andhra Pradesh	India	N. 18° 5′ 34.764″ E. 83° 8′ 10.557”	2014	Freshwater	MT040848
*Frontonia paramagna*	GVMC17	Mudasarlova Garden, Visakhapatnam, Andhra Predesh	India	N. 17° 45′ 43.175″ E. 83° 17′ 47.302”	2014	Freshwater	MT040849
*Frontonia vesiculosa*	KP2	Gosthani River near Kasipatnam, Andhra Pradesh	India	N. 18° 13′ 1.747″ E. 83° 6′ 41.954”	2014	Freshwater	MT040850

Seq. ID: accession number of 18S rDNA sequence deposited on GenBank

^a^Morphologically close to *Frontonia vernalis* (IPSal+b)

Moreover, in the present study, we tried to fix the *Frontonia* type species issue, providing the neotypification of *F. leucas* based on the diagnosis of an Italian population. Finally, a guideline for an accurate morphological description of *Frontonia* species is proposed at the end of the Discussion section.

## Results

### Neotypification of *Frontonia vernalis* (Ehrenberg, 1833) Ehrenberg, 1838

Class Oligohymenophorea de Puytorac et al., 1974

Order Peniculida Fauré-Frémiet in Corliss (1956)

Family Frontoniidae Kahl, 1926

Genus *Frontonia* Ehrenberg 1838

***Frontonia vernalis***

1833 *Bursaria vernalis* N. sp. Ehrenberg, Abh. K. Preuss. Akad. Wiss., P. 235. Pl. III

1838 *Bursaria* (*Frontonia) vernalis* Ehrenberg, Infusionsthierchen, P. 325. Pl. XXXIV, Fig. VII,

1841 *Panophrys (Bursaria) vernalis* Dujardin, Histoire Naturelle Des Zoophytes., P. 493. Pl. 32, Fig. 7.

1889 *Frontonia leucas* Schewiakoff, Beitr. Kennt. Holotrich. Ciliat. P. 40. Fig. 58.

1896 *Frontonia leucas* synon. *vernalis* Schewiakoff, Org.Sistem. Infus. Auctor. P. 312. Pl. V, Fig. 113.

1922 *Frontonia leucas* Penard, Etude infusoir. douce P. 139. Fig. 29.

1931 *Frontonia vernalis* Kahl, Tierwelt Deutsch. I. Wimp. Ciliata P. 317.

1943 *Frontonia vernalis* Kahl, Infusor. Handbuch. Prakt. repr. Acta Protistol. 43 (suppl.) P. 55.

1986 *Frontonia vernalis* Berninger et al., J. Protozool 33, P. 557. Fig. 6.

1986 *Frontonia leucas* Dragesco, Dragesco-Kerneis, Cilies libr. l’Afrique intertropic. P.318. Fig. 81b

2010 *Frontonia vernalis* Esteban et al., Protist 161, P. 629. Fig. 1e.

#### Diagnosis

Size in vivo 250 × 125 μm on average, size after staining 218.3 × 99.5 μm on average; cytostome/body length ratio: 1/7; 120–145 somatic kineties; macronucleus (Ma): 75.9 × 40.7 μm in size; micronucleus (Mi): 3–9, compact-type, 5.3 × 3.4 μm in size; peniculi: 4 + 4 + 4; vestibular kineties (VKs): 3; postoral kineties (PKs): 6–7; paroral membrane (PM): single-rowed; contractile vacuole (CV): single, with 8–13 canals; pore of contractile vacuole (PCV): single, on the dorsal side; pre-suture is continue on the dorsal side; pigment granules are absent; cysts not detected; during swimming ciliate rotation mainly to the right and, with less frequently, to the left direction. Several hundreds of *Chlorella*-like organisms (4–6 μm in diameter each) borne in ciliate cytoplasm. Freshwater.

#### Neotype locality

The sampling site of the neotype population of *F. vernalis* (IPSal+b) is the permanent freshwater shallow small pond located along the Ligurian sea coastline close to the mouth of Serchio River (Parco Naturale di Migliarino San Rossore Massaciuccoli, Migliarino, Pisa district, Tuscany, Italy, N. 43°47′7.524″ E. 10°15′57.44″), sample № 7 (sampling date 1 February 2017; collector S. I. Fokin).

#### Neotype material

One neotype slide with silver nitrate stained neotype specimen (registration number: CAMUS_2020–1), indicated by a circle of ink on the coverslip, plus a paratype slide with permanent Feulgen stained specimens (registration number: CAMUS_2020–2) have been deposited in the collection of the “Museo di Storia Naturale e del Territorio dell’Università di Pisa” (Calci, Pisa, Italy).

#### Voucher material

The total genomic DNA of the species obtained from cells of the neotype population is available at the Department of Biology of the University of Pisa, Zoology-Anthropology Unit. The 18S rDNA sequence of *F. vernalis* results (population IPSal+b) 1708 bp long and is deposited in NCBI GenBank database under the accession number MT040840.

#### Occurrence and ecology

Probably, a population (FSPBb) of the same species has been detected also in the Russian site: the small permanent ditch, Old Peterhof, St. Petersburg district, Russia (N. 59°52′45.88′′ E. 29°51′37.224′′). Nevertheless, due to the lack of molecular data on the latter population, the species attribution for Russian *Frontonia* FSPBb remains uncertain.

#### Morphological description of neotype population of *Frontonia vernalis*

**Fig. 2:**
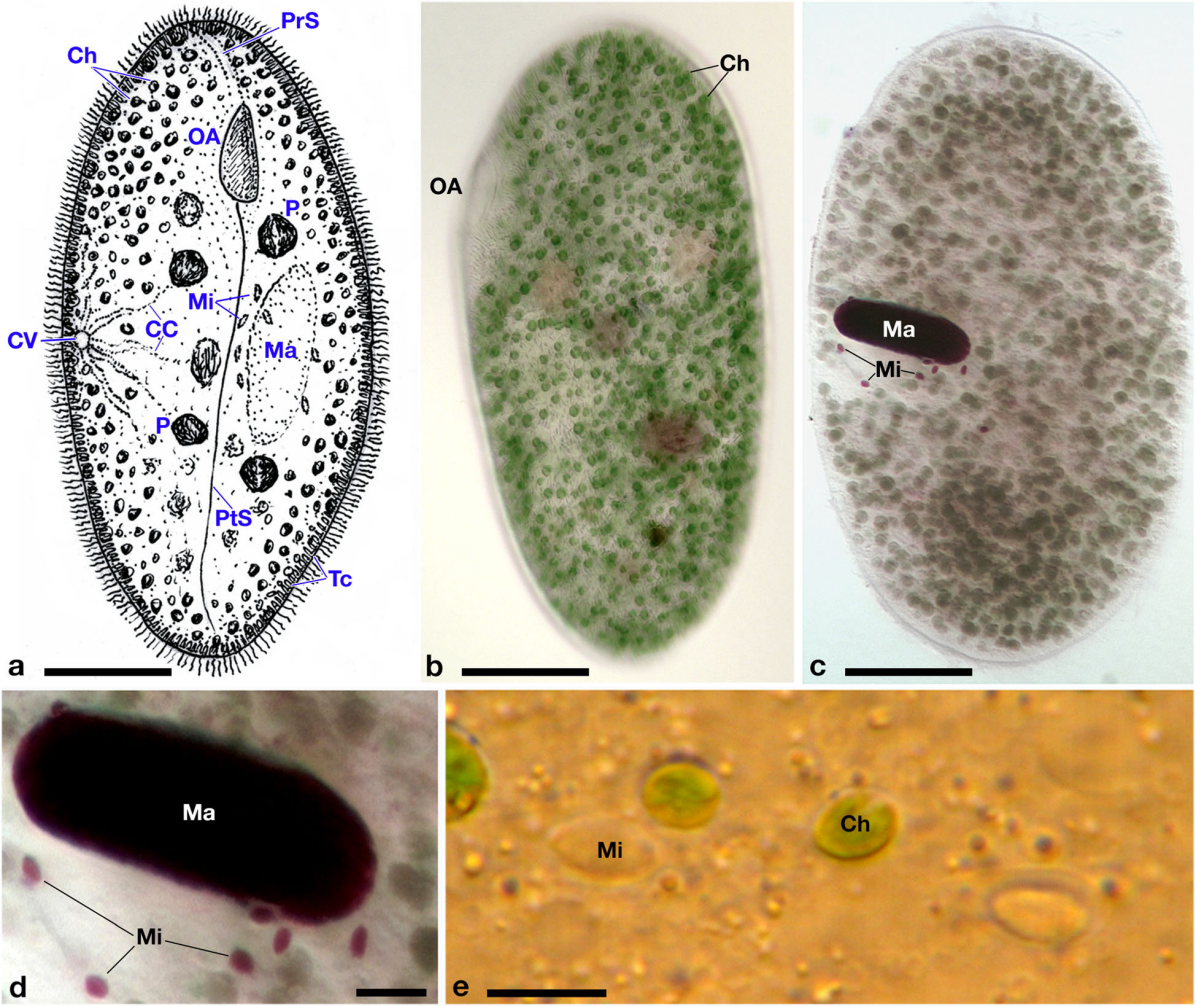
Morphology of *Frontonia vernalis* population IPSal + b (Neotype). **a** Schematic drawing of the ventral side; **b** live cell bearing *Chlorella*-like (Ch) endosymbiotic algae; **c** nuclear apparatus after Feulgen stainining, showing the macronucleus (Ma) and several micronuclei (Mi); **d** closer view of Ma and “compact type” Mi after Feulgen staining; **e** detail of Mi morphology and location in live cell, plus detail of *Chlorella*-like endosymbiont (Ch). CC – collecting canals; CV – contractile vacuole; OA – oral aperture; P – phagosomes; PrS – preoral suture, PtS – postoral suture; Tc – trichocysts. Bars stand for 50 μm (**a**-**c**), 10 μm (**d**), 5 μm (**e**)

**Fig. 3:**
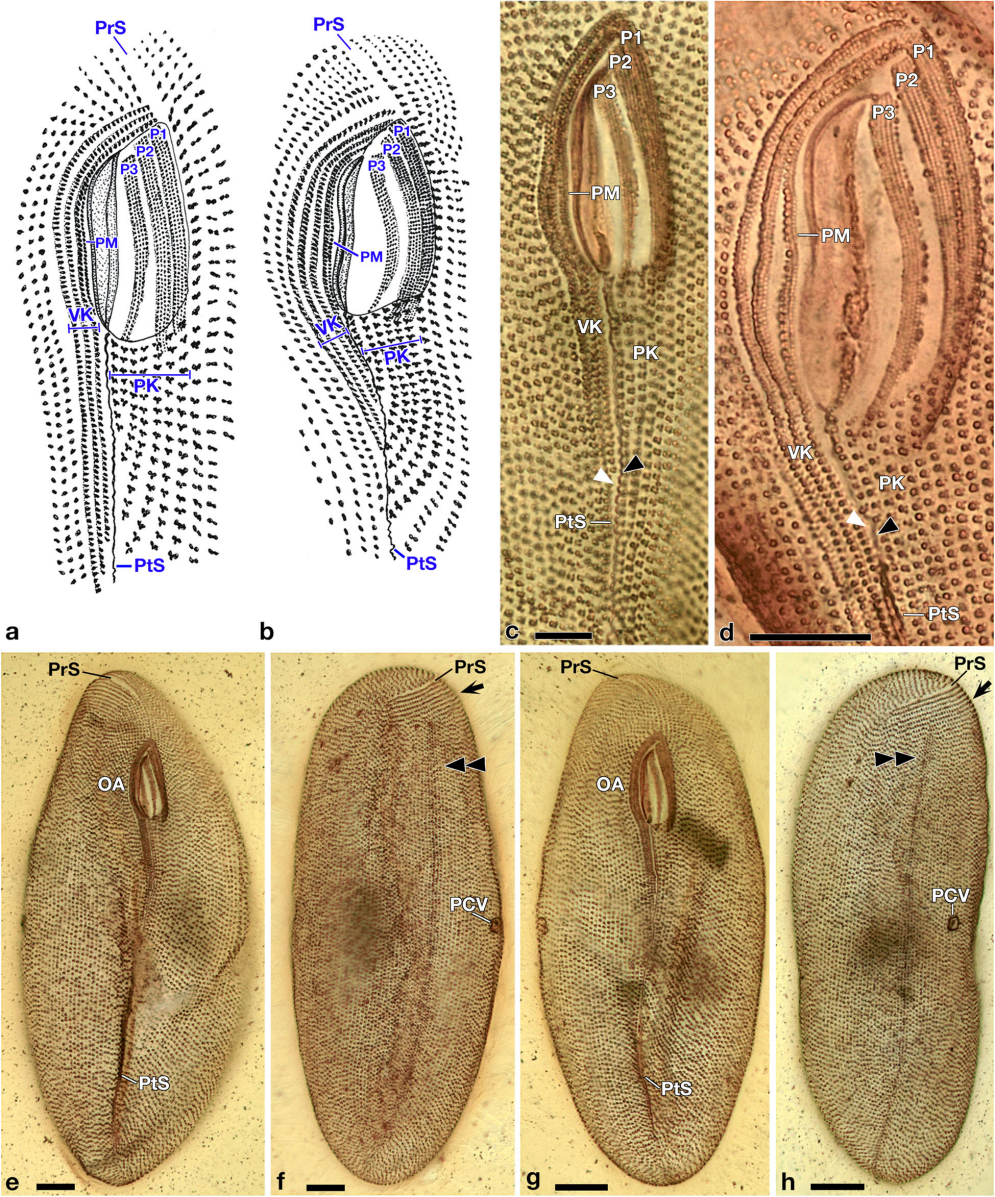
Oral and somatic ciliature of *Frontonia vernalis* (Neotype) (**a**, **c**, **e**, **f**) and *Frontonia paravernalis* sp. nov. (**b**, **d**, **g**, **h**) after silver impregnation. **a** Schematic drawing of oral apparatus of *F. vernalis*; **b** schematic drawing of oral apparatus of *F. paravernalis*; **c** closer view of *F. vernalis* oral ciliature and of the postoral suture (PtS), formed by a cortex folding (black arrowhead) and by an empty line, free of cilia (white arrowhead); **d** closer view of *F. paravernalis* oral ciliature and of the PtS; **e**, **f** ventral and dorsal somatic ciliature of *F. vernalis*; **g**, **h** ventral and dorsal somatic ciliature of *F. paravernalis*. OA – oral aperture; P1, P2, P3 - first, second, third peniculus; PCV - pore of contractile vacuole; PK – postoral kineties; PM – paroral membrane; PrS – preoral suture; PtS – postoral suture; VK – vestibular kineties; *Arrow* – dorsal set of kineties parallel to the preoral suture; *Black Arrowhead* – cortex folding, similar to a comb, forming part of the PtS; *Double Arrowhead* – longitudinal dorsal stripe of adjacent kineties; *White Arrowhead* – cortex line, free of cilia, forming part of the PtS. Bars stand for 20 μm (**e**-**h**), 10 μm (**c**, **d**)

Italian population, IPSal+b (Figs. 2 and 3). Cell shape ovoid with a broadly rounded anterior end and a narrower, rounded posterior end in vivo (Fig. 2a, b). Cells dorso-ventrally flattened. Size about 220–300 × 120–140 μm in vivo (250 × 125 μm on average). Silver stained ciliates shorter (on average): 218.3 × 99.5 μm (Table 2). Length:width ratio close to 2:1. Somatic cilia about 10 μm long; caudal cilia sometimes slightly longer. Meridional ciliary rows, around 120–145, visible in silver stained cells: 60–72 ventral, 60–73 dorsal (Fig. 3e, f; Table 2). Some ventral ciliary rows (especially in the posterior part of the left side) terminating before the end of the body, approaching the postoral suture. Postoral suture conspicuous, running almost to the posterior pole of the body, consisting of some empty spaces from the right side and twisted argentophilic fold in the pellicle from the left side of the structure (Fig. 3a, c, e). Cytoproct, blending into this fold, difficult to detect. On the dorsal side postoral suture not observed (Fig. 3f).

**Table 2:** Morphometric data on *Frontonia vernalis,* Serchio population (IPSal+b) - Neotype, Italy

Characters	$\overline{\mathrm{X}}$	SD	Min	Max	CV	n
Body, length^a^	218.3	16.4	183.0	247.0	7.51	20
Body, width^a^	99.5	14.3	73.3	120.0	14.4	20
Somatic ciliary rows, ventral side, number	66.7	3.9	60	72	5.8	12
Somatic ciliary rows, dorsal side, number	63.0	17.3	60	73	27.5	12
Excretory pores, number	1	0	1	1	0	15
Macronucleus, length^b^	75.9	8.0	62.0	85.5	10.5	18
Macronucleus, width^b^	40.7	3.9	35.0	45.3	9.6	18
Micronucleus, number	5.1	1.9	3	9	36.6	16
Micronucleus size, length^b^	5.3	0.7	4.0	7.5	14.7	18
Micronucleus size, width^b^	3.4	0.6	2.5	4.5	16.9	18

*X̅* Arithmetic mean, *SD* Standard deviation, *Min* Minimum, *Max* Maximum, *CV* Coefficient of variation in percentage, *n* Number of investigated cells

^a^Data based on Chatton–Lwoff silver-stained cells

^b^Macro- and micronuclei were measured from Feulgen-stained ciliates. Measurements in μm

Preoral suture presenting an empty space going on, from upper side of buccal aperture to the right-anterior part of dorsal side (Figs. 2a and 3f). In the dorsal side, several kinetosomal rows (8–10), beneath the preoral suture end, travelling parallelly to each other, with an angle of 45° respect with the longitudinal axis of cell (Fig. 3f). On the left, in the middle of dorsal side, 3–4 kineties tightly close to each other, forming a visible longitudinal strip in the kinetome (Fig. 3f).

Basal bodies on the ventral side (around the oral region) consisting of dikinetids, in the dorsal side consisting of monokinetids (Fig. 3a, c). Kinetosomes of oral region forming triplets after impregnation: dikinetids + parasomal sacs (Fig. 3a, c).

Buccal apparatus, 1/7 of body length, about 32 μm, located on the ventral side, about 18% back from anterior body end (Figs. 2a and 3a, c, e). Formed by three symmetrical, almost parallel and slightly curved peniculi on the buccal left side, composed of four rows of basal bodies each (Fig. 3a, c; Table 2). Peniculi showing (I + II + III) 4 + 4 + 4 rows of cilia, with peniculus III presenting 1–2 rows of cilia in its posterior end (Fig. 3a, c); from the right side, the buccal cavity presenting a single-rowed PM, closely associated with the first VK (Fig. 3a, c). Three VKs, gradually elongating posteriorly from left to right (Fig. 3a, c). PKs, 6–7, gradually shortening from the left posterior angle of buccal cavity to postoral suture (Fig. 3a, c, e). Single CV with almost straight 8–13 collecting canals, not always well-visible, with a single PCV opening on the right part of the dorsal side, in the equator of the cell (Fig. 2a). PCV usually well visible, rounded, occupying the space of 3–4 ciliary rows (Fig. 3f).

Numerous resting spindle-shaped extrusomes (trichocysts), about 8 μm long and 1.5 μm wide, with a conical arrowhead-like tip, and a rounded cross-section; similar to those of *Paramecium* or majority of other frontoniids (Fig. 2a). Extruded organelles about 10–11 times the length of those in resting state, resembling transparent spines. Ma in mid-body position; always ellipsoidal, 62.0–85.5 × 35.0–45.3 μm in size, after Feulgen staining (Fig. 2c, d; Table 2). Several compact-type Mi (3–9; in average 5), 5.3 × 3.4 μm in size after Feulgen staining, usually located close to the Ma (Fig. 5c, d; Table 2).

Despite the morphology of the Russian population (FSPBb) appears consistent with that of the Italian population (IPSal+b), without supporting molecular data we must be cautious, avoiding referring to it as *F. vernalis*.

#### Endosymbionts and other inclusions

Endosymbiotic *Chlorella*-like algae were present in the cytoplasm of *F. vernalis* (see Results and Discussion sections). No additional symbionts other than cytoplasmic green algae were observed either in the cytoplasm or in the nuclear apparatus of the ciliates. Some inclusions of different size and unknown nature, and food vacuoles mainly containing bacteria, diatoms, and dinoflagellates were usually present in the cytoplasm of ciliates, freshly isolated from native population (Fig. 2b, e).

#### Biology

In the native environment, cells of *F. vernalis* appeared mainly concentrated on the surface or slightly beneath of bottom sediments, but they also occurred in pelagic part of the water column. The ciliate preferably swam rotating to the right, but sometimes it could switch to the left spiral as well, with respect to the longitudinal body axis. Resting cysts not observed. Apparently, this frontoniid ciliate species could also eat rotifers and some other ciliates (preferably *Euplotes*). We did not succeed to keep the ciliate in culture using the dinoflagellate *Peridinium* sp. as food as indicated by UK colleagues, who cultivated the species they referred to as “*F. vernalis*” in the laboratory [33].

#### Molecular identification

The 18S rDNA sequence of *F. vernalis* IPSal+b (MT040840) showed the highest identity with sequences of *F. shii* (MF279208) and “*F. vernalis*” (U97110): 99.3% (ten mismatches) and 99.2% (one gap, 23 mismatches), respectively (Table 3).

**Table 3:** Identity values among green frontoniids and selected *Frontonia* 18S rDNA sequences

		a.	b.	c.	d.	e.	f.	g.	h.	i.	j.	k.	l.	m.	n.	o.	p.	q.	r.
a.	***Frontonia paramagna***^**a**^	100																	
b.	*Frontonia paramagna* MF279207	100	100																
c.	*Frontonia paramagna* JQ868786	99.9	100	100															
d.	***Frontonia vesiculosa*** **(KP2)**	99.4	99.4	99.4	100														
e.	***Frontonia*** **sp. (VmFr)**	98.5	98.6	98.5	98.6	100													
f.	***Frontonia*** **sp. (IPSal-)**	98.5	98.6	98.5	98.6	100	100												
g.	*Frontonia* sp. AF255359	98.7	98.6	98.6	98.6	99.8	99.8	100											
**h.**	***Frontonia paravernalis*** **(IPSal + sm)**	**98.4**	**98.4**	**98.3**	**98**	**98**	**98**	**98.2**	**100**										
**i.**	***Frontonia paravernalis*** **(FSPBsm)**	**98.4**	**98.4**	**98.3**	**98**	**98**	**98**	**98.2**	**100**	**100**									
j.	“*Frontonia vernali*s” U97110	98.5	98.6	98.5	98.2	98.2	98.2	98.2	**99**	**99**	100								
k.	*Frontonia shii* MF279208	98.6	98.6	98.6	98.2	98.2	98.2	98.3	**99.1**	**99.1**	99.2	100							
**l.**	***Frontonia vernalis*** **(IPSal + b) - Neotype**	**98.8**	**98.8**	**98.8**	**98.6**	**98.4**	**98.4**	**98.5**	**99**	**99**	**99.2**	**99.3**	**100**						
m.	Uncultured ciliate EU910593	97.5	97.5	97.5	97.3	97.5	97.5	97.6	**97.7**	**97.7**	97.5	97.6	**97.7**	100					
n.	“Frontonia angusta” (?) MG456580	96.9	96.9	96.9	96.9	96.5	96.5	96.6	**96.9**	**96.9**	96.6	96.7	**96.9**	97.7	100				
o.	*Frontonia leucas* (IPBG) AM072622	97.1	97.1	97	97.1	96.6	96.6	96.8	**97.1**	**97.1**	96.8	96.8	**97.1**	97.8	99.9	100			
p.	***Frontonia leucas*** **(KNP3)**	97.1	97.1	97	97.1	96.6	96.6	96.8	**97.1**	**97.1**	96.7	96.8	**97.1**	98.1	99.4	99.5	100		
q.	Uncultured ciliate EU910594	97.1	97.1	97	97.1	96.6	96.6	96.8	**97.1**	**97.1**	96.7	96.8	**97.2**	97.7	99.2	99.3	99.5	100	
r.	*Frontonia* sp. KJ475307	96.7	96.7	96.6	96.8	96.6	96.6	96.8	**96.8**	**96.8**	96.5	96.7	**96.9**	97.4	97.8	97.8	98	97.9	100

Identity values obtained via distance matrix calculation by ARB program; sequences obtained in present work are shown in *bold*

^a^*Frontonia paramagna* ML, KTC4, KKR19, BDM3, KT1, GVMC17 populations

### Description of *Frontonia paravernalis* sp. nov.

Class Oligohymenophorea de Puytorac et al., 1974

Order Peniculida Fauré-Frémiet in Corliss (1956)

Family Frontoniidae Kahl, 1926

Genus *Frontonia* Ehrenberg, 1838

***Frontonia paravernalis*** **sp. nov.**

#### Diagnosis

Size in vivo 178 × 95 μm on average, size after staining 159.0 × 90.2 μm on average; length:width ratio 1.8:1; cytostome/body length ratio: 1/6; 98–116 somatic kineties; Ma: 48.5 × 23.2 μm in size; Mi:1–3 (usually 2), compact-type, 3.4 × 2.6 μm in size; peniculi: 4 + 4 + 4; VKs: 3–4 (mainly 4); PKs: 5–7; PM: single-rowed; CV: single, with 8–11 canals; PCV: single, on the dorsal side (around 4.5 μm in diameter); pre-suture continuing on dorsal side; pigment granules absent; no cysts found; during swimming, cell rotation mainly to the right, and less frequently to the left. Several hundreds of *Chlorella*-like organisms (4–6 μm in diameter) borne in cell cytoplasm. Freshwater.

#### Type locality

The sampling site of the type population of *F. paravernalis* (IPSal+sm) is the permanent freshwater shallow small pond located along the Ligurian sea coastline close to the mouth of Serchio River (Parco Naturale di Migliarino San Rossore Massaciuccoli, Migliarino, Pisa district, Tuscany, Italy, N. 43° 47′ 7.524″ E. 10° 15′ 57.44″), sample № 7 (sampling date 1 February 2017; collector Fokin).

#### Type material

One holotype slide with silver nitrate stained holotype specimen (registration number: CAMUS_2020–3), indicated by a circle of ink on the coverslip, plus a paratype slide with permanent Feulgen stained specimens (registration number: CAMUS_2020–4) have been deposited in the collection of the “Museo di Storia Naturale e del Territorio dell’Università di Pisa” (Calci, Pisa, Italy).

#### Etymology

“Paravernalis”, *para,* “beside; next to, near” from Ancient Greek *pará* (παρά); *vernalis*, from the specific epithet of *F. vernalis*, the first green *Frontonia* ever described which the novel species has many traits in common with.

#### Voucher material

The total genomic DNA of the species obtained from cells of the type population is available at the Department of Biology of the University of Pisa, Zoology-Anthropology Unit. The 18S rDNA sequences of *F. paravernalis* resulted 1707 bp long and were deposited in NCBI GenBank database under the accession numbers MT040839 (population IPSal+sm), and MT040838 (population FSPBsm).

#### Occurrence and ecology

The same species has been detected in Russia, in a small permanent ditch in Old Peterhof (St. Petersburg district, Russia, N. 59° 52′ 45.88′′ E. 29° 51′ 37.224′′), sample № 3 (sampling date 16 August 2016; collector Fokin).

#### Morphological description of type population

**Fig. 4:**
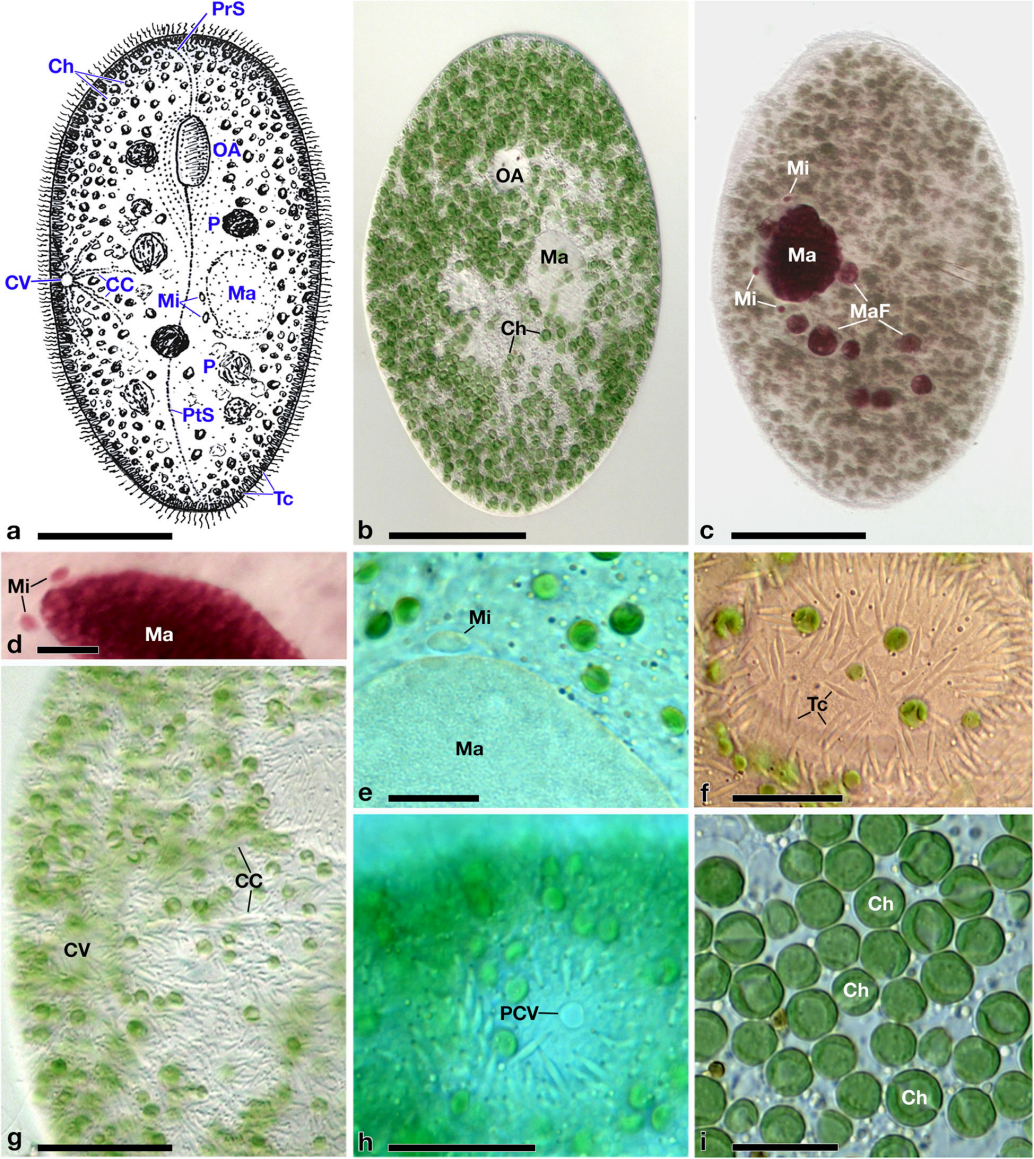
Morphology of *Frontonia paravernalis* sp. nov (Italian population - IPSal + sm). **a** Schematic drawing of the ventral side; **b** live cell bearing *Chlorella*-like (Ch) endosymbiotic algae; **c** nuclear apparatus after Feulgen stainining, showing the macronucleus (Ma), several micronuclei (Mi) and the old macronuclear fragments (MaF); **d** closer view of Ma and “compact type” Mi after Feulgen staining; **e** detail of Mi morphology and location in living condition; **f** detail of trichocysts (Tc) in living cell; **g** closer view of contractile vacuoles (CV) and collecting canals (CC); **h** detail of the single pore of contractile vacuole (PCV) in living condition; **i** detail of *Chlorella*-like endosymbionts (Ch) after *F. paravernalis* cell disruption. CC – collecting canals; Ch – *Chlorella*-like endosymbiont; CV – contractile vacuole; Ma – macronucleus; MaF – macronuclear fragment; Mi – micronucleus; OA – oral aperture; P – phagosomes; PCV – pore of contractile vacuole; PrS – preoral suture, PtS – postoral suture; Tc – trichocysts. Bars stand for 50 μm (**a**-**c**), 20 μm (**f**-**h**), 10 μm (**d**, **i**), 5 μm (**e**)

**Fig. 5:**
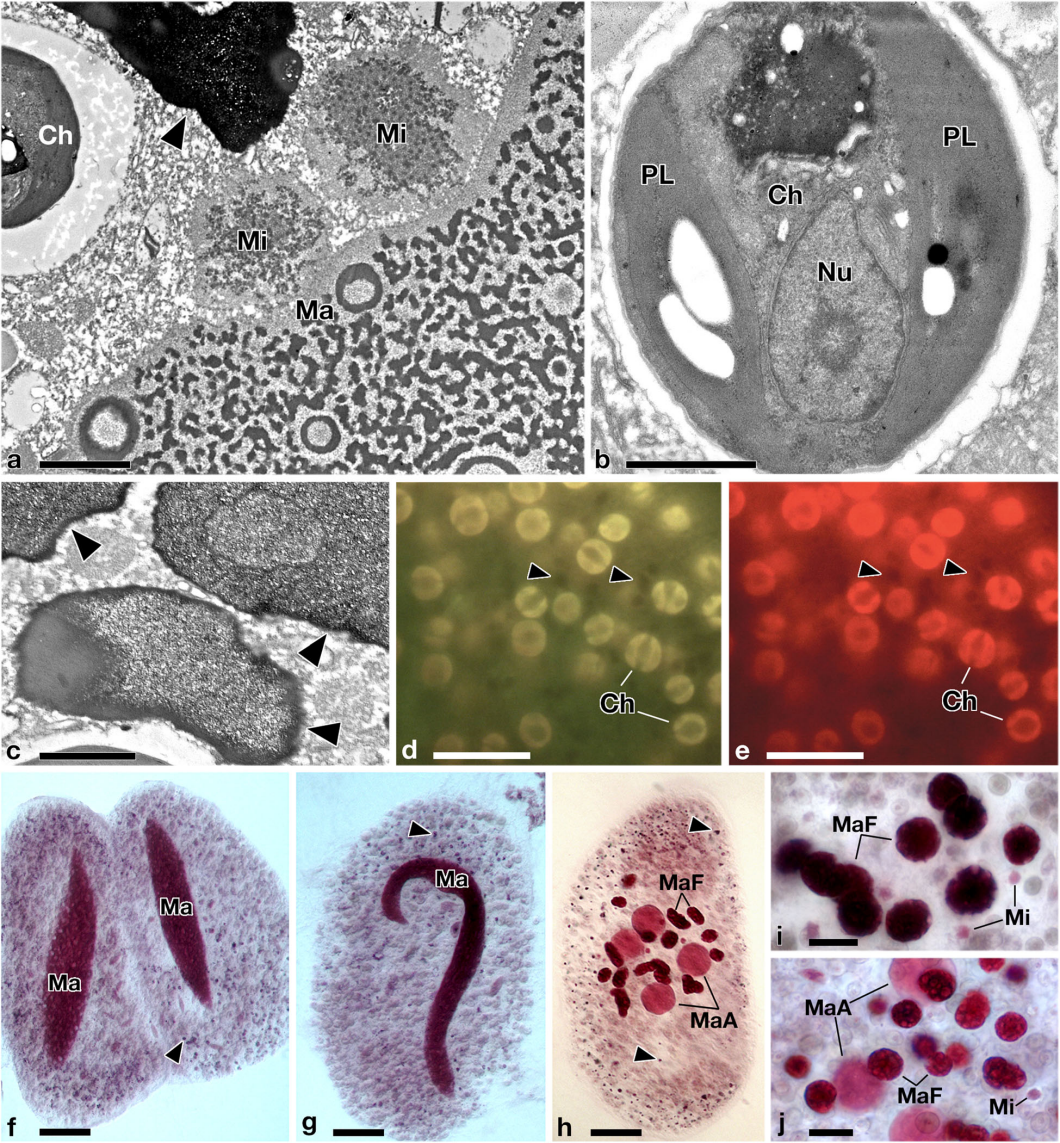
TEM (**a**-**c**) and FISH images (**d**, **e**), and conjugation process after Feulgen staining (**f**-**j**) of *Frontonia paravernalis* sp. nov. **a** Detail of macronucleus (Ma), micronucleus (Mi), *Chlorella*-like endosymbiont (Ch), and unknown particle (arrowhead); **b** closer view of *Chlorella*-like endosymbiont (Ch); **c** detail of unknown particles (arrowhead); **d**, **e** FISH images showing autofluorescent *Chlorella*-like endosymbionts (Ch) and unknown particles (double arrowhead) negative to eubacterial (**d**) and alphaproteobacterial (**e**) probes; **f** conjugant pair; **g** exconjugant with still not macronucleus (Ma); **h** exconjugant with new macronuclear anlagen (MaA) and old macronuclear fragments (MaF); **i** closer view of MaF and micronuclei (Mi); **j** detail of new MaA, MaF, and Mi. Ch – *Chlorella*-like endosymbiont; Ma – macronucleus; MaA – macronuclear anlagen; MaF – macronuclear fragments; Mi – micronucleus; Nu – nucleus; PL – plastid; *Arrowhead* – unknown particle. Bars stand for 25 μm (**f**-**h**), 10 μm (**d**, **e**), 5 μm (**i**, **j**), 1 μm (**a**-**c**)

Italian population, IPSal+sm (Figs. 3, 4 and 5). Cell shape ovoid with rounded anterior and posterior ends in vivo (Fig. 4a, b). Cells slightly dorso-ventrally flattened. Size about 140–190 × 80–100 μm in vivo (average 178 × 95 μm). Silver stained ciliates are shorter: 159.0 × 90.2 μm on average (Table 4). Length:width ratio close to 1.8:1. Somatic cilia about 10 μm long; caudal cilia sometimes slightly longer. Meridional ciliary rows 98–116, visible in silver stained cells: 48–58 ventral, 50–58 dorsal (Fig. 3g, h; Table 4). Some of the ventral ciliary rows (especially in the posterior part of the left side) terminating before the end of body, approaching the postoral suture. Suture very conspicuous, running almost to the posterior pole of the body, consisting of some little empty space from the right side and twisted argentophilic fold in the pellicle from the left side of the structure. Cytoproct, blending into this fold, difficult to detect. On the dorsal side, postoral suture not observed (Fig. 3h). Preoral suture consisting of as an empty space going on from the upper side of buccal aperture to the right-anterior part of the dorsal side (Figs. 3b, g and 4a). In the dorsal side several kinetosomal rows (6–8), beneath the preoral suture end, travelling parallelly to each other, with an angle of 45° with respect to the longitudinal axis of cell (Fig. 3h). On the left, in the middle of dorsal side, 2–3 kineties tightly close to each other, forming a visible longitudinal strip in the kinetome (Fig. 3h). Basal bodies on the ventral side (around the oral region) consisting of dikinetids, in the dorsal consisting of monokinetids (Fig. 3b, d). Kinetosome units of oral region forming triplets after silver staining: dikinetids + parasomal sacs (Fig. 3b, d).

**Table 4:** Morphometric data on *Frontonia paravernalis* sp. nov.

Characters	$\overline{\mathrm{X}}$	SD	Min	Max	CV	n
Body, length^a^	**159.0**	**21.6**	**142.0**	**170.0**	**13.6**	**33**
	171.7	10.1	152.0	183.0	5.9	15
Body, width^a^	**90.2**	**12.5**	**80.0**	**102.2**	**13.8**	**23**
	100.4	6.0	93.3	112.0	6.0	15
Somatic ciliary rows, ventral side, number	**52.7**	**3.7**	**48**	**58**	**7.0**	**14**
	46.2	5.4	35	56	11.6	10
Somatic ciliary rows, dorsal side, number	**52.5**	**2.6**	**50**	**58**	**4.9**	**14**
	48.8	3.6	42	57	7.4	10
Excretory pores, number	**1.1**	**0.3**	**1**	**2**	**27.2**	**20**
	1.0	0	1	1	0	20
Macronucleus, length^b^	**48.5**	**5.5**	**35.0**	**60.0**	**11.3**	**17**
	52.3	5.2	45.0	60.0	9.9	12
Macronucleus, width^b^	**23.2**	**3.9**	**17.3**	**29.2**	**16.6**	**17**
	28.4	5.0	20.0	40.0	17.6	12
Micronucleus, number	**2.2**	**0.8**	**1**	**3**	**35.5**	**38**
	2.3	0.6	1	3	27.8	10
Micronucleus, length^b^	**3.4**	**0.6**	**2.5**	**5.0**	**17.1**	**18**
	3.9	1.2	3.0	6.5	32.4	10
Micronucleus, width^b^	**2.6**	**0.2**	**2.3**	**3.0**	**8.2**	**18**
	2.5	0.3	2.5	3.0	12.6	10

Morphometric data for the type population of *Frontonia paravernalis* sp. nov. from Serchio Italy (IPSal + sm) are shown in bold (upper row). Morphometric data for *F. paravernalis* population from Peterhof, Russia (FSPBsm), are shown in normal font (lower row)

*X̅* Arithmetic mean, *SD* Standard deviation, *Min* Minimum, *Max* Maximum, *CV* Coefficient of variation in percentage, *n* Number of investigated cells

^a^Data based on Chatton–Lwoff silver-stained cells

^b^Macro- and micronuclei were measured from Feulgen-stained ciliates. Measurements in μm

Buccal apparatus, 1/6 of body length, about 28 μm, located on the ventral side, about 12% back from anterior body end (Figs. 3g and 4a). Formed by three symmetrical, almost parallel and slightly curved peniculi on the buccal left side, composed of four rows of basal bodies each (Fig. 3b, d; Table 4). Peniculi showing (I + II + III) 4 + 4 + 4 rows of cilia, with peniculus III presenting 2–3 rows of cilia in its posterior end (Fig. 3b, d; Table 4); from the right side, buccal cavity presenting a single-rowed PM closely associated with the first vestibular kinety (Fig. 3b, d). VKs, 3–4 (mainly four), gradually elongating posteriorly from left to right (Fig. 3b, d, g). PKs, 5–7, gradually elongated from postoral suture to the left posterior angle of buccal cavity (Fig. 3b, d, g). Single CV with almost straight 8–11 collecting canals, with a single PCV opening on the right part of the dorsal side, in the equator of the cell (Fig. 4a, b, g; Table 4). PCV relatively large and rounded (around 4.5 μm in diameter), occupying the space of 3–4 ciliary rows (Figs. 3h and 4h).

Numerous resting spindle-shaped extrusomes (trichocysts), about 8 μm long and 1.4 μm wide, with conical, arrowhead-like tip, and a rounded cross section, similar to those of *Paramecium* or to those already described in *Frontonia* [42] (Fig. 4a, f). Extruded organelles measuring about ten times the length of those in resting state, resembling transparent spines. Mid body-located, slightly ellipsoidal Ma, 35.0–60.0 × 17.3–29.2 μm in size after Feulgen staining (Fig. 4a, c-d; Table 4). One to three compact-type Mi (usually two), 3.4 × 2.6 μm in size after Feulgen staining, usually located close to the Ma (Figs. 4a, c-d and 5a; Table 4).

#### Diagnosis of Russian population (FSPBsm)

Size in vivo 190 × 105 μm on average; size after stainig 171.7 × 100.4 μm on average; length:width ratio 1.7:1; cytostome/body length ratio: 1/6; 77–113 somatic kineties. Ma: 52.3 × 28.4 μm in size; Mi: 1–3 (usually 2), compact-type, 3.9 × 2.5 μm in size. Peniculi: 4 + 4 + 4; VKs: 3–4; PKs: 5–7. CV: single, with 8-11canals; PCV: one in the dorsal side (around 4.5 μm in diameter); pre-suture continuing on dorsal side; pigment granules absent; no cysts found; during swimming, cell rotation mainly to the right, and less frequently to the left. Several hundreds of *Chlorella*-like organisms (4–6 μm in diameter) borne in cell cytoplasm. Freshwater (Suppl. Fig. 1; Table 4).

#### Endosymbionts and other inclusions

Endosymbiotic *Chlorella*-like organisms (4–6 μm in diameter) were detected in the cytoplasm of Italian and Russian frontoniids (Fig. 4a, b, i). Number of the endosymbiontic algae (about several hundred) varied from cell to cell; symbionts are mainly located below the cortical layer, but they could be detected in endoplasm as well (Figs. 4a, b, i and 5b). A certain divergence among the 18S rDNA sequences of *Chlorella*-like organisms from Italian and Russian frontoniids, suggests that different species of algae colonize *F. paravernalis* cells, depending on the sampling site (see further Results and Discussion sections).

Some other inclusions of different size and nature such as food vacuoles with bacteria, diatoms and dinoflagellates were usually present in the cytoplasm of ciliates freshly isolated from native population.

Besides the stained nuclear apparatus and *Chlorella*-algae nuclei, in many cells of the Italian population of *F. paravernalis* treated for Feulgen reaction, a number of positive particles with variable size (0.5–2.0 μm) were observed. They were distributed in the cytoplasm, first of all beneath cortex in the anterior and posterior ends of the ciliate (Fig. 4a, b). Apparently, the same inclusions were visible in TEM sections (Fig. 5a, c), where they appear encircled by a membrane and show inner structures delimited by membranes as well. These inclusions did not resemble bacteria according to their morphology and did not show positive signal to *alphaproteobacterial* and *eubacterial* probes during fluorescence in situ hybridization (FISH) (Fig. 5d, e), leading to the hypothesis that they were not prokaryotic endosymbionts.

#### Biology

In the native environment cells of *F. paravernalis* appeared mainly concentrated on the surface or slightly beneath of bottom sediments but were always present in pelagic zone as well. In both populations (Italian and Russian), ciliates mainly swam rotating to the right with respect to the longitudinal body axis; rarely a switch to the left spiral rotation was observed. Under laboratory conditions the Italian population cells showed no clear light-positive reactivity. Resting cysts were not observed. We did not succeed to keep the ciliate in culture using the dinoflagellate *Peridinium* sp. as food as indicated by UK colleagues, who cultivated the species they referred to as “*F. vernalis*” in the laboratory [33].

In IPSal+sm population of *F. paravernalis* conjugation process was repeatedly observed under laboratory conditions. As conjugated pairs for cytological investigation were isolated from population, we have no knowledge of the mating type system of the species. However, the main steps of the sexual process were investigated using Feulgen stained preparations (Fig. 5f-j). Three progamic divisions of Mi with crescent stage were revealed. Then, the new nuclear apparatus was rebuilt after three metagamic divisions in which from synkaryon usually developed 4 Ma (sometimes 5–6) anlagens as well as 4 Mi. The fragmentation process of the old Ma usually started pretty late, after separation of partners and even after the first synkaryon division. Before the process, the old Ma changed its shape to very characteristic spindle-like form and, after separation of the partners, to twisted-sausage shape. The old Ma was fragmented usually into about 8–18 fragments (Fig. 5h-j).

#### Molecular identification

The 18S rDNA sequences of *F. paravernalis* [MT040839 (population IPSal+sm), and MT040838 (population FSPBsm)] resulted identical among each other and showed the highest identity with sequences of *F. shii* (MF279208) and “*F. vernalis*” (U97110): 99.1 and 99.0%, respectively (Table 3). Moreover, they showed 99.0% identity with *F. vernalis* from present study (one gap and 15 mismatches).

### Neotypification of *Frontonia leucas* based on IPBG population from Italy

***Frontonia leucas*** **(Ehrenberg, 1833) Ehrenberg, 1838**

#### Diagnosis based on the IPBG population from Pisa (Italy)

Size after silver staining 130–210 × 60–95 μm; cytostome/body length ratio: 1/7; 98–110 somatic kineties; Ma: 35–50 × 15–40 μm in size; Mi: 2–3, compact-type, 3.0–5.0 × 2.5–4 μm in size; peniculi: 5 + 5 + 5; VKs: 3; PKs: 5–6; PM: single-rowed; CV: single, with 8–11 canals; PCV: single, large; pigment granules absent; no cysts found; swimming rotation mainly to the right (only sometimes to the left). Freshwater.

#### Neotype locality

Freshwater pond in Botanical Garden of University of Pisa, Pisa, Italy (N. 43°43′ 10.97″ E. 10°23′45.387″).

#### Neotype material

The slide with the silver-stained neotype specimen (indicated with a black circle of ink on the coverslip) has been deposited in the collection of the ‘Museo di Storia Naturale dell’Università di Pisa’ (Calci, Pisa, Italy), with registration number CAMUS_2020–5.

#### Voucher material

The 18S rDNA sequence of *F. leucas* resulted 1631 bp long and was already deposited in NCBI GenBank database under the accession number AM072622 [9]. It showed the highest identity with the sequences of “*F. angusta”* (MG456580) and *F. leucas* from India (KY855558): 99.9 and 99.5% respectively (Table 3).

#### Morphological description of neotype population of *Frontonia leucas*

Italian population, IPBG (Supplementary Figure 2). Cell shape ovoid with both rounded anterior and posterior end in vivo (Suppl. Fig. 2a, b). Cells dorso-ventrally flattened. Size 220 × 95 μm on average μm in vivo. Size after silver staining 130–210 × 60–95 μm. Length:width ratio close to 2:1. Somatic cilia about 10 μm long; caudal cilia sometimes slightly longer. Somatic ciliary rows, around 98–110, visible in silver stained cells (Suppl. Fig. 2e, f). Some ventral ciliary rows (especially in the posterior part of the left side) terminating before the end of the body, approaching the postoral suture. Postoral suture conspicuous, running almost to the posterior pole of the body, consisting of some empty spaces from the right side and twisted argentophilic fold in the pellicle from the left side. Cytoproct, blending into this fold, difficult to detect. On the dorsal side, not visible postoral suture (Suppl. Fig. 2f).

Preoral suture presenting an empty space going on, from upper side of buccal aperture to the right-anterior part of dorsal side (Suppl. Fig. 2f, g). In the dorsal side, several kinetosomal rows (5–6), beneath the preoral suture end, travelling parallelly to each other, with an angle of 45° respect with the longitudinal axis of cell (Suppl. Fig. 2f, g). On the left, in the middle of dorsal side, 2–3 kineties tightly close to each other, forming a visible longitudinal strip in the kinetome (Suppl. Fig. 2f).

Basal bodies: on the ventral side (around the oral region) consisting of dikinetids; in the dorsal side consisting of monokinetids. Kinetosomes of oral region forming triplets after impregnation: dikinetids + parasomal sacs.

Buccal apparatus, 1/7 of body length, about 28 μm, located on the ventral side, about 15% back from anterior body end (Suppl. Fig. 2a, e). Peniculi showing (I + II + III) 5 + 5 + 5 rows of cilia (Suppl. Fig. 2h); from the right side, the buccal cavity presenting a single-rowed PM, closely associated with the first VK (Suppl. Fig. 2h). Three VKs, gradually elongating posteriorly from left to right (Suppl. Fig. 2h). PKs, 5–6, gradually shortening from the left posterior angle of buccal cavity to postoral suture (Suppl. Fig. 2h). Single CV with 8–11 long and twisted collecting canals, situated in the right part of the dorsal side, in the equator of the cell (Suppl. Fig. 2f).

Numerous resting spindle-shaped extrusomes (trichocysts), about 8 μm long and 1.5 μm wide, with a conical arrowhead-like tip, and a rounded cross-section; similar to those of *Paramecium* or majority of other frontoniids. Ma in mid-body position; always ellipsoidal, 35–50 × 15–40 μm in size, after Feulgen staining (Suppl. Fig. 2b, d). Two to three compact-type Mi (commonly three) 3.0–5.0 × 2.5–4 μm in size after Feulgen staining, usually located close to the Ma (Suppl. Fig. 2c, d). Pigment granules absent. Resting cysts not observed. Swimming rotation mainly to the right, rarely to the left, with respect to the longitudinal body axis.

### Diagnosis of other retrieved frontoniids

#### *Frontonia leucas* population KNP3 (Kolleru Lake, Andhra Pradesh, India, freshwater)

Size after silver staining 134–191 × 99–157 μm; cytostome/body length ratio: 1/6; 90–108 somatic kineties; Ma: 25–36 × 11–29 μm in size; Mi: 2–4, compact-type, 1.5–2.0 μm in size; peniculi: 5 + 5 + 5; VKs: 3; PKs: 6; CV: single, with 7–8 canals; PCV: 1, large; no pigment granules; no cysts found; during swimming it can rotate in both directions, but mainly to the left. The 18S rDNA sequence of *F. leucas* resulted 1707 bp long and was deposited in NCBI GenBank database under the accession number (KY855558). It showed the highest identity with sequences of *F. leucas* (AM072622), and “*F. angusta”* (MG456580): 99.5, and 99.4%, respectively (Table 3).

#### Material deposition

Slide “UNIPI_2020–1” with Feulgen-stained specimens (indicated with black circles of ink on the coverslip). The slide has been deposited in the collection of the Anthropology-Zoology Unit, Department of Biology, University of Pisa, (Pisa, Italy).

#### *Frontonia paramagna* populations KTC4 and KKR19 (Kolleru Lake, Andhra Pradesh, India, freshwater)

Size after silver staining 270.2–322.0 × 121.8–158.9 μm; cytostome/body length ratio: 1/10; 166–208 somatic kineties; Ma: 63.8 × 30.6 μm in size; Mi: 6–14, compact-type, 1.5–2.3 μm in size; peniculi: 4 + 4 + 4; VKs: 3; PKs: 6–7; CV: 1–4, with 10–14 canals; PCV: 1–3; no pigment granules; no cysts found; during swimming it can rotate in both directions (i.e., left and right). The 18S rDNA sequences of *F. paramagna* populations KTC4 and KKR19 resulted 1709 bp and 1708 bp long, respectively, and were deposited in NCBI GenBank database under the accession numbers KY855557 and KY855554, respectively. They showed the highest identity with the sequences of *F. paramagna*-MF279207 and *F. paramagna*-JQ868786 (type sequence of the species): 100 and 99.9%, respectively (Table 3). Identical sequences were obtained from frontoniid populations BDM3, KT1, and GVMC17 sampled in India (deposited in NCBI GenBank database under the accession numbers: MT040847, MT040848, MT040849, respectively), although no morphological data are available for them.

#### Material deposition

Slide “UNIPI_2020–2” with silver-stained voucher specimens (indicated with black circles of ink on the coverslip). Slide “UNIPI_2020–3” with Feulgen-stained voucher specimens (indicated with black circles of ink on the coverslip). Slides have been deposited in the collection of the Anthropology-Zoology Unit, Department of Biology, University of Pisa, (Pisa, Italy).

#### *Frontonia vesiculosa* population KP2 (Gosthani River, Andhra Pradesh, India, freshwater)

Size after silver staining 450–700 × 200–250 μm; cytostome/body length ratio: 1/10; 150–180 somatic kineties; Ma: 70–90 × 30–50 μm in size; Mi: 8–12, compact-type, 3.5–4.5 μm in size; peniculi: 5 + 5 + 5; VKs: 3–4; PKs: 6–7; CV: 7–11, with 12–15 canals; PCV: 1–2; permanent pigment granules absent; no cysts found; during swimming it can rotate in both directions (i.e., left and right). The 18S rDNA sequence of *F. vesiculosa* resulted 1708 bp long and was deposited in NCBI GenBank database under the accession number (MT040850). It showed the highest identity with sequences of *F. paramagna* (MF279207, JQ868786, and those from present study): 99.4% (Table 3).

#### *Frontonia* sp. population IPSal- (Serchio River, Italy, freshwater)

Size after silver staining 145–200 × 70–105 μm; cytostome/body length ratio: 1/8; 92–105 somatic kineties; Ma: 30–45 × 15–30 μm in size; Mi:1–3, compact-type, 3.0–3.2 μm in size; peniculi: 5 + 5 + 5; VKs: 3; PKs: 6–7; CV: single, with 9–11 canals; PCV: one in the dorsal side; pre-suture is continue on the dorsal side; no pigment granules; no cysts found; during swimming it mainly rotates to the right, and less frequently to left direction. The 18S rDNA sequence of *Frontonia* sp. IPSal- resulted 1708 bp long and was deposited in NCBI GenBank database under the accession number (MT040841). It showed the highest identity with the sequence of *Frontonia* sp. (AF255359): 99.8% (Table 3). An identical sequence was obtained from *Frontonia* sp. population VmFr, sampled in Sardinia (Monte Urpino Park, Cagliari, Italy) (deposited in NCBI GenBank database under the accession number MT040842), although no morphological data are available for this population.

#### *Frontonia atra* population F4 (Serchio River, Italy, freshwater)

Size after silver staining 80–100 × 40–50 μm; cytostome/body length ratio: 1/4; 80–90 somatic kineties; Ma: 15–25 × 15–20 μm in size; Mi: 1–2, vesicular-type, 2.5–3.5 μm in size; peniculi: 4 + 4 + 3; VKs: 4; PKs: 4; CV: single, without canals; PCV: 2–4; pigment granules present; no cysts found. The 18S rDNA sequence of *F. atra* resulted 1663 bp long and was deposited in NCBI GenBank database under the accession number (MT040844). It showed the highest identity with sequences of *F. minuta* (MT040846) and an uncultured ciliate (AY821929): 98.6 and 98.2%, respectively (Table 5).

**Table 5:** Identity values among selected *Frontonia* 18S rDNA sequences

		a.	b.	c.	d.	e.	f.	g.	h.	i.	j.	k.	l.	m.	n.	o.	p.	q.	r.	s.	t.	u.	v.	w.	x.	y.	z.	aa.	ab.	ac.
a.	*Frontonia sinica* KJ475308	100																												
**b.**	***Frontonia*** **sp. (BJ4)**	**98.2**	**100**																											
c.	*Frontonia magna* FJ868199	98.3	**98.2**	100																										
d.	*Frontonia salmastra* MH319376	98.1	**98.3**	98.6	100																									
e.	*Frontonia subtropica* FJ868202	98.2	**98.3**	98.7	98.9	100																								
f.	*Frontonia canadensis* KJ475312	97.3	**97.9**	97.9	98.3	98.5	100																							
g.	*Frontonia lynni* DQ190463	94.8	**94.8**	94.8	95.1	94.7	94.9	100																						
h.	*Frontonia tchibisovae* KJ475316	94.4	**94.4**	94.3	94.7	94.4	94.4	97.6	100																					
i.	*Frontonia mengi* FJ875141	94.6	**94.5**	94.7	94.7	94.8	94.7	95.5	95.2	100																				
j.	*Frontonia didieri* KJ475298	91.7	**91.8**	91.9	92.2	92	92.3	91.7	91.3	91.9	100																			
**k.**	***Frontonia fusca*** **(F3)**	**91.5**	**91.9**	**91.7**	**92**	**91.8**	**91.9**	**91.2**	**90.8**	**91.5**	**97.7**	**100**																		
l.	*Frontonia ocularis* FJ868198	91.4	**91.7**	91.6	91.9	91.7	91.9	91.3	91	91.7	97.9	**99.7**	100																	
m.	*Frontonia elegans* KJ475301	92.2	**92.1**	92.4	92.5	92.5	92.4	91.5	91.1	92.1	98.7	**97.9**	98	100																
n.	*Frontonia anatolica* MG456578	91.9	**91.8**	92.3	92.2	92.1	92.1	91.3	91	91.8	97.5	**97**	97.1	97.7	100															
o.	*Frontonia pusilla* FJ868201	91.5	**91.7**	91.7	91.8	91.9	91.4	90.8	90.5	91.4	96.6	**96.2**	96	97.1	96.8	100														
p.	*Apofrontonia dohrni* AM072621	91.4	**91.5**	91.9	91.8	92	91.8	90.8	90.5	91.4	95	**94.8**	94.7	95.4	94.7	95	100													
q.	*Paramecium tetraurelia* AB252008	89	**89.2**	89.3	89.7	89.4	89.4	88.6	88.5	88.8	91.8	**91.2**	91.2	91.7	91.6	90.8	90.5	100												
r.	*Paranassula* sp. FJ998039	90.5	**90**	90.5	90.4	90.5	90.8	89.6	89.1	89.6	90.7	**89.9**	90.1	90.9	90.5	89.9	89.9	88.1	100											
**s.**	***Frontonia minuta*** **(F2)**	**91.3**	**91.3**	**91.3**	**91.4**	**91.1**	**91.2**	**90.9**	**90.6**	**91.1**	**92**	**91.9**	**92.2**	**92**	**91.6**	**91.1**	**90.8**	**90.3**	**90.3**	**100**										
t.	Uncultured ciliate AY821929	91.3	**91.1**	91.3	91.4	91.1	91.1	91.1	90.6	91.2	92.2	**92**	92.4	92.1	91.9	91.2	91	90.1	90.3	**99.5**	100									
**u.**	***Frontonia atra*** **(F4)**	**91.3**	**91.5**	**91.3**	**91.3**	**91.2**	**91.1**	**90.7**	**90.4**	**91.1**	**91.8**	**91.6**	**91.9**	**91.9**	**91.6**	**91.2**	**90.8**	**90.4**	**90.4**	**98.6**	**98.2**	**100**								
v.	Frontoniidae sp. LN870026	91	**91**	91.3	91.4	91.1	90.9	90.8	90.4	91.3	92.3	**92.2**	92.3	92.6	92.2	91.3	91.4	91	89.8	**96.3**	96.2	**96.3**	100							
w.	*Frontonia acuminata* MG456579	90.7	**90.8**	91.1	91.1	90.9	90.7	90.6	90.2	91.2	92.2	**92**	92.1	92.4	92	91.1	91.3	90.8	89.5	**95.9**	95.9	**95.9**	99.6	100						
x.	*Frontonia terricola* MF926593	91.6	**91.6**	92.1	92.1	92.1	91.7	91.1	91	92.3	92.1	**92.2**	92.3	92.5	92.5	91.7	91.3	89.8	89.3	**94.7**	94.7	**94.5**	95.8	95.5	100					
y.	Frontoniidae sp. LN869925	91.9	**91.5**	91.6	91.9	91.7	91.5	91.1	91	91.5	91.6	**91.6**	91.7	91.8	91.3	91.5	91.9	89.7	89.6	**93.1**	93.1	**92.4**	93.5	93.2	93.8	100				
z.	*Marituja* cf. *caudata* MF926594	91.4	**91.2**	91.4	91.4	91.4	91	91.5	91.3	91.5	91.6	**91.8**	91.9	92.2	92	91.1	91.2	90	89.5	**93.4**	93.4	**92.9**	93.8	93.4	94.5	93.2	100			
aa.	*Disematostoma minor* MF926592	91.6	**91.5**	91.8	91.8	91.9	91.5	91.9	92	92	91.3	**91.6**	91.6	91.7	92.5	91.1	91.4	90.2	89.1	**92.9**	92.9	**92.3**	92.9	92.6	94.2	93.5	97.7	100		
ab.	*Stokesia vernalis* HM030738	91.2	**91.2**	91.5	91.5	91.6	90.9	90.8	90.8	91.4	91.5	**91.4**	91.6	91.8	91.7	91.1	91.2	90	88.9	**93.3**	93.3	**92.9**	93.5	93.2	94.1	93	95.6	95.5	100	
ac.	*Lembadion bullinum* AF255358	90	**90**	90	90.4	90.1	90	89.2	89.4	89.8	89.3	**89**	89	89.8	89.7	89.4	89.7	87.7	88.1	**89.2**	89.2	**89**	89.8	89.7	90.3	90.3	90	89.9	89.9	100

Identity values obtained via distance matrix calculation by ARB program; sequence obtained in present work is shown in *bold*

#### *Frontonia minuta* population F2 (Serchio River, Italy, freshwater)

Size after silver staining 60–90 × 40–60 μm; cytostome/body length ratio: 1/5; 50–65 somatic kineties; Ma: 15–20 × 10–15 μm in size; Mi: two, endosomal-type, 1.5–2.0 μm in size; peniculi: 4 + 4 + 3; VKs: 3–4; PKs: 3–4; CV: single, without canals; PCV: 2–4; no pigment granules; extrusomes relatively long with respect to ciliate size; no cysts found. The 18S rDNA sequence of *F. minuta* resulted 1663 bp long and was deposited in NCBI GenBank database under the accession number (MT040846). It showed the highest identity with sequences of an uncultured ciliate (AY821929) and *F. atra* (MT040844): 99.5 and 98.6%, respectively (Table 5).

#### *Frontonia fusca* population F3 (Serchio River mouth, Italy, brackish water: salinity 14‰)

Size after silver-staining 90–150 × 45–70; cytostome/body length ratio: 1/5; 75–92 somatic kineties; Ma: 25–35 × 20–25 μm in size; Mi: 1–2, endosomal-type, 1.5–2.0 μm in size; peniculi: 4 + 4 + 3; VKs: 3; PKs: 4; PM: double-rowed; CV: two, with 6–9 canals; PCV: 2–3, on the dorsal side; pigment granules are present as a spot on the anterior right point of dorso-lateral side; no cysts found; pre- and postoral sutures widely run on the dorsal side; during swimming it preferably rotates to the right direction [10]. The 18S rDNA sequence of *F. fusca* resulted 1667 bp long and was deposited in NCBI GenBank database under the accession number (MT040845). It showed the highest identity with the sequence of “*F. ocularis”* (FJ868198): 99.7% (Table 5). The close identity and morphological affinity with “*F. ocularis*” will be further discussed below.

#### *Frontonia* sp. population BJ4 (India, brackish water: salinity 5‰)

Size after silver staining 180–250 × 100–150 μm; cytostome/body length ratio: 1/5; 113–134 somatic kineties; Ma: 45–70 × 20–40 μm in size; Mi: 1, compact-type, 4.3–4.6 μm in size; peniculi: 4 + 4 + 4–3; VKs: 4–5; PKs: 4–5; PM: double-rowed; CV: single, with a net of collecting canals; PCV one in the dorsal side; no pigment granules; extrusomes rhombic in section; pre- and postoral sutures widely run on the dorsal side; no cysts found; during swimming it rotates to the left direction. The 18S rDNA sequence of *Frontonia* sp. BJ4 resulted 1711 bp long and was deposited in NCBI GenBank database under the accession number (MT040843). It showed the highest identity with sequences of *F. subtropica* and *F. salmastra* (98.3%), *F. sinica*, and *F. magna* (98.2%) (Table 5).

### Molecular phylogeny of *Frontonia*

**Fig. 6:**
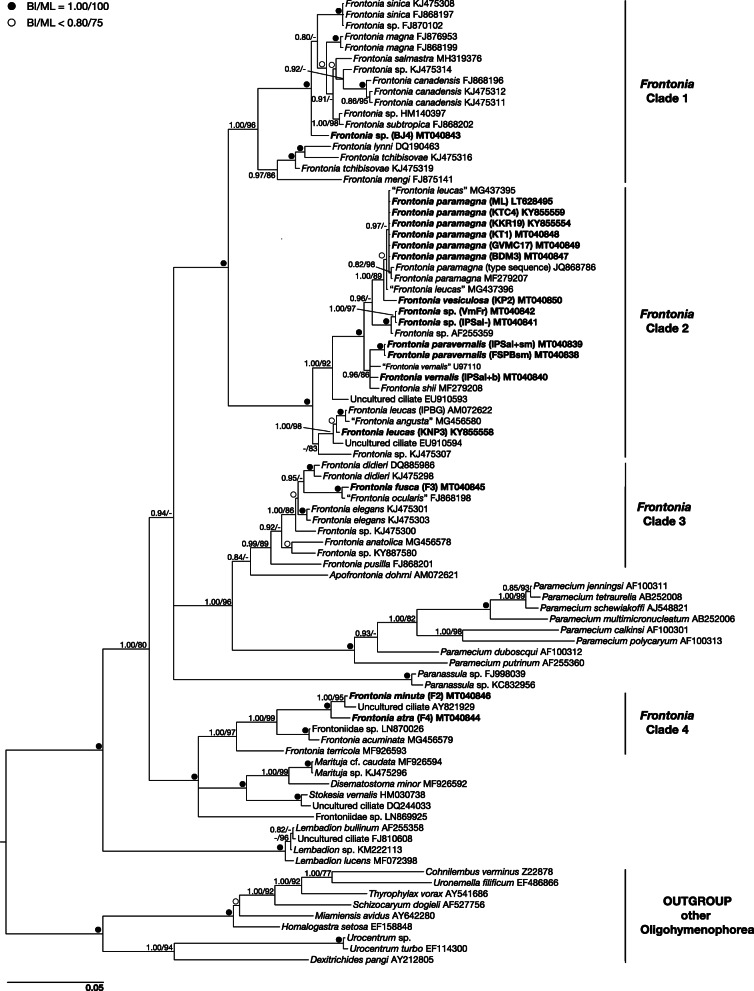
Phylogenetic tree of the subclass Peniculia based on 18S rDNA sequences. Phylogenetic relationships of *Frontonia vernalis* (neotype) and *Frontonia paravernalis* sp. nov.: they cluster in the same clade of *“F. vernalis”* and *F. shii*. Phylogenetic position of the other *Frontonia* spp. in analysis is shown as well. Genus *Frontonia* resulted paraphyletic, forming four different clades (Clade1–4). Numbers associated to nodes represent posterior probability from Bayesian inference (BI) and bootstrap value from maximum likelyhood (ML) analyses, respectively (only values of BI > 0.80 and ML > 75% are shown). *Black dots* represent the highest statistical support (BI = 1.00 and ML = 100); *white dots* indicate non-significant statistical support (BI < 0.80 and ML < 75). Sequences obtained in the present work are in *bold*

Phylogenetic relationships of *Frontonia* species presented in this study are reported in Fig. 6. As for the *F. vernalis* and the novel species from the present study, the topology of BI/ML tree showed that they cluster in the same clade of “*F. vernalis”* (U97110) and *F. shii* (MF279208) with quite high statistical support (0.96/86). Unfortunately, the phylogenetic relationships inside the clade were not resolved by the analysis, showing polytomies.

The genus *Frontonia* appeared to be not monophyletic. Four clades can be distinguished, with the following species composition: Clade 1: *F. sinica, F. magna, F. salmastra, F. subtropica, F. canadensis, F. tchibisovae, F. lynni, F. mengi,* and *Frontonia* spp.; Clade 2: *F. paramagna, F. vesiculosa, “F. vernalis”, F. shii, F. vernalis* (present study)*, F. paravernalis, F. leucas, “F. angusta”* and *Frontonia* spp.; Clade 3: *F. didieri, “F. ocularis”, F. elegans, F. fusca, F. anatolica,* and *F. pusilla;* Clade 4: *F. acuminata, F. minuta, F. atra, F. terricola,* and uncultured frontoniids (AY821929, LN870026, LN869925).

Clade 1 and Clade 2 resulted sister clades, forming a monophyletic group with high statistical support, i.e., 1.00/100. In the molecular tree, the position of the sequences of “*F. leucas”* (MG437395–96) in Clade 1, and “*F. angusta”* (MG456580) in Clade 2, is questionable and will be later discussed, since the species attribution made by Kizildag and Yildiz [25] in our opinion should be revised (i.e., the morphological description of these two species diverges from the original descriptions of *F. leucas* sensu Ehrenberg and Foissner and *F. angusta* sensu Foissner).

Clade 3 appeared sister of *Apofrontonia dohrni*, and they (Clade 3 + *Apofrontonia*) together resulted sister group of *Paramecium* (1.00/96).

Clade 4 grouped together with sequences of *Marituja, Disematostoma, Stokesia*: in this group, evolutionary relationships were not resolved, showing polytomies.

Peniculia group members (*Frontonia, Apofrontonia, Paramecium, Paranassula, Stokesia*, and *Lembadion*) clustered together showing high values of statistical support (1.00/100).

The sequence of *Frontonia* sp. BJ4, branched basally to *F. sinica* – *F. magna – F. salmastra – F. canadiensis – F. subtropica* – clade, with high statistical support (1.00/100), inside Clade 1.

Sequences of *F. paramagna* (LT628495, KY855554, KY855559, MT040847–49) clustered with conspecifics, including the type sequence of the species (JQ868786), inside Clade 2. Since sequences under the name “*F. leucas*” (MG437395–6) clustered in the same *F. paramagna*-clade (significantly far from the type sequence of *F. leucas* – AM072622), we strongly believe that those organisms were misidentified (see Discussion section).

Sequence from *F. vesiculosa* (MT040850) resulted the sister species of *F. paramagna*.

Sequences from IPSal- and VmFr *Frontonia* sp. clustered together with *Frontonia* sp.-AF255359, forming a clade sister to *F. paramagna* + *F. vesiculosa*. These two populations (IPSal- and VmFr) constitute, to the best of our knowledge, a novel *Frontonia* species.

Sequence of *F. leucas* KNP3 (KY855558) branched basally to the type sequence of *F. leucas* (AM072622). In this clade, the sequence indicated as “*Frontonia angusta*” (MG456580), clustered with the type sequence of *F. leucas*: in our opinion some error or misidentification occurred (see Discussion section).

*Frontonia fusca*’s sequence (MT040845) clusters together with *F. ocularis* sequence, in Clade 3. Given the morphological and phylogenetic affinity between the two organisms we are prone to consider them as the same species, thus following the Principle of Priority of the ICZN (Article 23) we keep as valid *F. fusca* (Quennerstedt, 1869) Kahl, 1931. Consequently, *F. ocularis* described by Bullington [31] and redescribed by Pan and colleagues [14] should be treated as the junior synonym of *F. fusca* ([17], this study) and indicated at least as “*F. ocularis*”.

Sequences of *F. minuta* and *F. atra* clustered in Clade 4, being sister of *F. acuminata* and an uncultured frontoniid (LN870026).

### Phylogeny of endosymbionts of *F. vernalis* and *F. paravernalis* sp. nov.

The 18S rDNA sequence of the endosymbionts from the Italian population of *F. vernalis* (IPSal+b) resulted 1796 bp long and showed a high identity with several species of the family *Chlorellaceae*: 100% with *Chlorella* sp. (X72706), 99.9% with *Chlorella sorokiniana* (FM205834), 99.8% with *Micractinium reisseri* (endosymbiont of *P. bursaria*) (AB437244) (Table 6). It was deposited in NCBI GenBank under the accession number MT040853.

**Table 6:** Identity values among *Frontonia* endosymbionts and selected 18S rDNA sequences

		a.	b.	c.	d.	e.	f.	g.	h.	i.	j.	k.	l.	m.	n.	o.	p.	q.	r.	s.	t.	u.	v.	w.	x.
a.	*Meyerella planktonica* AY195973	100																							
**b.**	**Endosymbiont of** ***F. paravernalis*** **from Russia (FSPBsm)**	**99.4**	**100**																						
c.	*Chlorella* sp. symbiont of *Hydra* AB713410	99.4	**99.4**	100																					
d.	*Chlorella* sp. symbiont of *Hydra* AB713408	99.5	**99.4**	100	100																				
e.	*Chlorella variabilis* from *Paramecium bursaria* AB260893	99	**98.9**	99	99.1	100																			
f.	*Chlorella variabilis* from *P. bursaria* AB219527	98.9	**98.8**	99	99	100	100																		
g.	*Lobosphaeropsis lobophora* X63504	98.8	**98.8**	98.8	98.8	99.4	99.3	100																	
h.	*Chlorella volutis* HQ111434	99	**98.8**	99.1	99.1	99.4	99.4	99.3	100																
i.	*Chlorella singularis* HQ111435	98.9	**99**	99.2	99.3	99.6	99.5	99.4	99.8	100															
j.	*Actinastrum hantzschii* FM205841	98.8	**98.9**	99.1	99.2	99.5	99.4	99.4	99.7	99.8	100														
k.	*Diacanthos belenophorus* AY323837	98.8	**98.9**	99.3	99.3	99.5	99.4	99.4	99.7	99.8	99.9	100													
l.	*Chlorella thermophila* KF661334	98.3	**98.4**	98.6	98.7	99	99	99	99.2	99.3	99.3	99.3	100												
m.	*Chlorella sorokiniana* FM205834	99	**99**	99.3	99.3	99.5	99.4	99.4	99.7	99.8	99.8	99.8	99.3	100											
n.	*Chlorella lewinii* FM205861	99	**99**	99.3	99.3	99.5	99.4	99.4	99.7	99.8	99.8	99.8	99.4	99.9	100										
o.	*Chlorella vulgaris* AB162910	98.7	**98.6**	98.8	98.9	99.2	99.1	99.4	99.4	99.5	99.4	99.4	99.1	99.6	99.4	100									
p.	*Chlorella pituita* GQ176853	98.4	**98.5**	98.8	98.9	99.1	99	99.2	99.3	99.4	99.3	99.4	99	99.4	99.3	99.6	100								
q.	*Micractinium reisseri* from *P. bursaria* AB437244	98.9	**99**	99.2	99.3	99.4	99.4	99.3	99.6	99.8	99.7	99.7	99.3	99.8	99.8	99.5	99.4	100							
r.	*Micractinium pusillum* AF237662	98.7	**98.7**	99	99	99.2	99.1	99.1	99.4	99.5	99.4	99.4	99.1	99.6	99.4	99.3	99.1	99.6	100						
**s.**	**Endosymbiont of** ***F. vernalis*** **from Italy (IPSal + b)**	**98.8**	**98.9**	**99.1**	**99.2**	**99.5**	**99.4**	**99.4**	**99.7**	**99.8**	**99.8**	**99.8**	**99.3**	**99.9**	**99.8**	**99.6**	**99.4**	**99.8**	**99.6**	**100**					
**t.**	**Endosymbiont of** ***F. paravernalis*** **from Italy (IPSal + sm)**	**98.8**	**98.9**	**99.1**	**99.2**	**99.5**	**99.4**	**99.4**	**99.7**	**99.8**	**99.8**	**99.8**	**99.3**	**99.9**	**99.8**	**99.6**	**99.4**	**99.8**	**99.6**	**99.9**	**100**				
u.	*Chlorella* sp. X72706	98.9	**99**	99.2	99.3	99.6	99.5	99.4	99.8	99.9	99.8	99.8	99.3	100	99.8	99.6	99.5	99.9	99.6	**100**	**100**	100			
v.	*Micractinium pusillum* FM205869	98.5	**98.8**	98.8	98.9	99.2	99.1	99.1	99.4	99.5	99.4	99.4	99.1	99.4	99.4	99.1	99.3	99.4	99.3	**99.4**	**99.4**	99.5	100		
w.	*Micractinium pusillum* AF364102	98.7	**98.7**	99	99	99.3	99.3	99.2	99.5	99.6	99.6	99.6	99.2	99.7	99.6	99.4	99.4	99.6	99.5	**99.7**	**99.7**	99.8	99.8	100	
x.	*Didymogenes anomala* FM205839	98.3	**98.3**	98.6	98.6	99	98.9	98.8	99.1	99.3	99.2	99.2	98.8	99.3	99.3	99	98.9	99.4	99	**99.3**	**99.3**	99.4	99.1	99.4	100

Identity values obtained via distance matrix calculation by ARB program; sequence obtained in present work is shown in *bold*

The 18S rDNA sequence of the endosymbionts of the Italian population of *F. paravernalis* (IPSal+sm) resulted 1796 bp long and identical to the one from endosymbionts of IPSal+b *F. vernalis* (Table 6). Interestingly, the same species of *Frontonia* from Russia (FSPBsm) harboured a different kind of green endosymbiont: their 18S rDNA sequences, 1758 bp long, showed the highest identity, 99.4%, with *Meyerella planktonica* (AY195973) and *Chlorella* sp. symbiont of *Hydra* sp. (AB713410, AB713408). The identity with the sequences of endosymbionts of Italian *F. paravernalis* resulted 98.9% (Table 6). If compared with the 18S rDNA sequence from the green endosymbiont of *P. chlorelligerum* (KX669637) found in the same sampling site in St. Petersburg [41], our endosymbiont’s sequence showed an identity of 99.0% (that sequence was not used in our phylogenetic reconstruction because too short). The 18S rDNA sequences of endosymbionts of *F. paravernalis* were deposited in NCBI GenBank under the accession numbers MT040852 (population IPSal+sm from Italy), MT040851 (population FSPBsm from Russia).

**Fig. 7:**
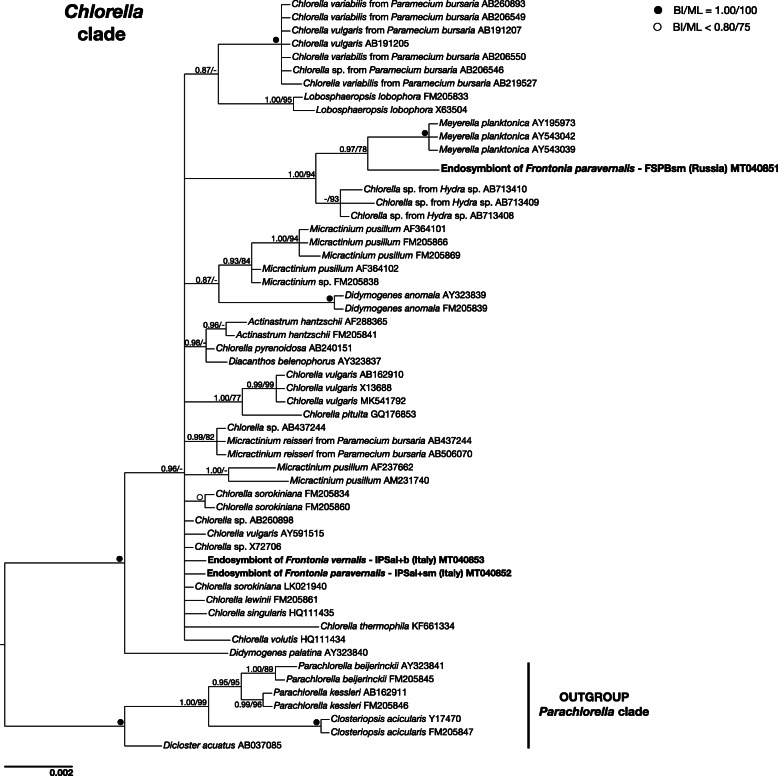
Phylogenetic tree of the *Chlorella* clade based on 18S rDNA sequences. Phylogenetic relationships of endosymbionts from Italian and Russian population of *Frontonia paravernalis* sp. nov. (IPSal+sm, FSPBsm) and from Italian population of *Frontonia vernalis* (IPSal+b) are shown. Endosymbionts from the same *Frontonia* species (IPSal+sm, FSPBsm - Italian and Russian *F. paravernalis*) cluster relatively far from each other, instead endosymbionts from the same site (Italy) cluster together, although they are hosted by different species (*F. paravernalis* - IPSal+sm and *F. vernalis* - IPSal+b). Numbers associated to nodes represent posterior probability from Bayesian inference (BI) and bootstrap value from maximum likelyhood (ML) analyses, respectively (only values of BI > 0.80 and ML > 75% are shown). *Black dots* represent the highest statistical support (BI = 1.00 and ML = 100); *white dots* indicate non-significant statistical support (BI < 0.80 and ML < 75). Sequences obtained in the present work are in *bold*

In our phylogenetic analysis the 18S rDNA sequences of endosymbionts from Italian populations of *Frontonia* (*F. vernalis* IPSal+b, *F. paravernalis* IPSal+sm) clustered significantly far respect with those from the Russian population (*F. paravernalis* FSPBsm) (Fig. 7). Indeed, endosymbionts from Italian frontoniids resulted close to some species of *Chlorella*, such as *C. sorokiniana* (LK021940), and *Chlorella* sp. (X72706), while endosymbionts from Russian *F. paravernalis* clustered with *Meyerella planktonica* (AY195973, AY543042, AY543039), with median statistical values (0.97/78). Moreover, sequences from symbionts of *Hydra* branched basally to the *Meyerella-Frontonia’*s endosymbiont-clade (statistical support: 1.00/94).

Beyond that, no other symbiotic *Chlorella*-like organisms did show any special phylogenetic affinity with our sequences. However, the phylogenetic relationships inside the *Chlorella*-clade were not completely resolved, and several polytomies occurred (Fig. 7). This might indicate a low-resolution power of the 18S rDNA as marker to study the phylogenetic relationships of this group of organisms.

## Discussion

### The *Frontonia vernalis* issue and its neotypyfication

Freshwater frontoniids hosting *Chlorella*-like cytoplasmic symbionts were repeatedly mentioned in the ciliatological literature since when Ehrenberg described one such ciliate under the name of *F. vernalis* [29, 30]. He found this species in the neighbourhood of Berlin and he gave the following diagnosis: “*Corpore ovato oblongo turgido, viridi, utrinque rotundato, postica parte paullo tenuiore, ore tertia quartave corporis parte superato*” [“Oval-oblong body, swollen, green, rounded at both ends, narrowed behind, mouth located in the apical third quarter of the body] [30]. He described this species as 211–254 μm long, carrying two CVs which, actually, together with the ciliate’s Ma, he misidentified as the male reproductive system of the “animal” (“*Eine grosse ovale männliche Sexualdrüse and 2 runde contractile Blasen bilden den männlichen Geschlechtsorganismus*” [The large oval male genital gland and two round contractile bladders form the male sexual apparatus]).

Later, Dujardin [43] mentioned *F. vernalis* (referred to as *Panophrys (Bursaria) vernalis*), however, he simply reported the description previously made by the German scientist. Thus, since Ehrenberg’s record, no other scientist was able to retrieve the ciliate corresponding to the descriptions made in 1833 and 1838. Although, later on, a green frontoniid was retrieved and identified as *F. vernalis* by UK researchers [33–35], its appearance was not completely fitting with that made by Ehrenberg (in particular it showed only a single CV vs. two CVs described by Ehrenberg). Unfortunately, our requests to UK colleagues to provide us with more precise information about the morphology of that *Frontonia* species (“*F. vernalis*”) have been ignored.

However, some doubts on the accuracy of Ehrenberg’s description can be expressed as well. For instance, Ehrenberg did not indicate the number of investigated cells. Moreover, in both publications (i.e., [29]: Plate III - Fig. IV, and [30]: Plate XXXIV - Fig. VII) he used the same illustrations, showing three different cells of *F. vernalis* (one of the cells appeared crushed in the version dated 1833). In the latest publication [30], the Author provided an additional image of *F. vernalis*, showing two larger specimens plus a significantly smaller third cell. Considering these three complete ciliate’s images (Fig. 1), two of them possess two CVs without canals (larger cells), while in the third cell only a single CV with canals is visible [29, 30]. Therefore, it is difficult to cope with such uncertain data about the number of CVs in the type description of *F. vernalis* (i.e., it is challenging to confidently address the question “does the species always possess two CVs?”).

It must be stressed that the Author illustrated a conjugating couple ([29]: Plate III-Fig IV [30];: Plate XXXIV, Fig VII) (Fig. 1) describing it as a “dividing” specimen. In our opinion, according to his statements [“*Einige Thierchen fand ich in der Längstheilung begriffen*”: “I found some animals in the process of longitudinal division”] he probably never observed a division process in the retrieved green frontoniids, which actually takes place along the transverse cell axis as typical of ciliates. Very likely, Ehrenberg saw some specimens in a pre-dividing phase (indeed, he selected two large cells), although without recognizing them as such. Given the fact that in peniculine ciliates CV duplicates before cell division, this could explain why Ehrenberg detected two of such organelles.

Therefore, we can hypothesize different scenarios about the description of *F. vernalis* and its real morphology: i) Ehrenberg detected a mixed population of green frontoniid species, one with two CVs plus another carrying only a single CV, i.e., *F. vernalis* possesses two CVs and has never been found again; ii) Ehrenberg detected a green frontoniid species which presented variability in its CV number, i.e., *F. vernalis* possesses either one or two CVs; iii) Ehrenberg described a green frontoniid species in a pre-division stage, i.e., *F. vernalis* possesses one CV and the description made by Ehrenberg should not be considered valid concerning this particular trait.

In our opinion, the first two hypotheses can be considered rather unlikely, because i) if existing, green *Frontonia* with two CVs should have been found again, at least another time; ii) variability of CV number in a *Frontonia* species is a quite rare event:the number of CVs is a stable feature for almost all frontoniids except for *F. vesiculosa, F. paramagna,* which present several CVs ([20], present study), and for *F. betica* [44] and *F. magna* [13], which may present one or two CVs.

In conclusion, after this careful consideration of all aspects, we are prone to consider more reliable the third hypothesis, and we propose the neotypification of *F. vernalis*, based on the green frontoniid retrieved in Italy (population IPSal+b). This species basically possesses all the (few) characteristics mentioned by Ehrenberg except the presence of two CVs: body size and shape are comparable (length: 220–300 μm vs. 211–254 μm), both species were retrieved in freshwater habitats and present *Chlorella*-like endosymbionts.

Moreover, the 18S rDNA sequence of the newly retrieved *F. vernalis* shows a high identity value with “*F. vernalis*” - U97110 sequence (Hirt and colleagues, 1997), although not being completely identical.

Unfortunately, it was not possible to clearly assess whether our *F. vernalis* and the species which the U97110 sequence belongs to are identical for the following reasons: i) a morphological description associated to this sequence is lacking; ii) the U97110 sequence presents many uncertain nucleotides (6 “N”, 1 “W”, 1 “Y”), which makes the comparison with our retrieved sequence not so reliable.

Consequently, since an exhaustive morphological description of the organism corresponding to the sequence U97110 (Hirt and colleagues, 1997) is lacking, the latter should be only carefully used for phylogenetic reconstruction and we recommend referring to it as *Frontonia* sp. or as “*F. vernalis*”*.*

### Comparison among green *Frontonia* species

Green frontoniids were repeatedly sampled by ciliatologists, but often misidentified (see Background and Discussion sections above). For example, the indication that *F. minuta* may also harbour *Chlorella-*like symbionts [20] sounds rather unexpected and has never been reported again. Therefore, to date, only few green *Frontonia* species were correctly identified. Beside some reports of *F. vernalis* sensu Ehrenberg, only one new *Frontonia* species bearing *Chlorella*-like endosymbionts was recently retrieved and described through a multidisciplinary characterization, i.e., *F. shii* [15].

The species treated in the present study, *F. vernalis* (neotype) and *F. paravernalis* sp. nov., present morphological and phylogenetic affinities with this recently described species (Table 7). These three green frontoniids cluster together in our phylogenetic analysis (Fig. 6), although molecular data and morphological features clearly indicate that *F. shii* does not coincide with any of our two target species.

**Table 7:** Comparative characterization of green frontoniids

Characters	***Frontonia vernalis*** sensu Ehrenberg	***F. vernalis*** (Neotype)	***F. paravernalis*** sp. nov.	***F. shii***
Average body length of living cells (μm)	233	250	178	300
Somatic kineties, number	ND	120–145	98–116	128–142
Contractile vacuoles, number	1–2	1	1	1
Collecting canals, number	ND	8–12	8–11	~ 10
PCV, number, position	ND	1, dorsal	1, dorsal	1, ventro-lateral
Postoral suture silver line	ND	Ends before the posterior end of the cell	Ends before the posterior end of the cell	Reach the posterior end of the cell
Peniculi (I + II + III)	ND	4 + 4 + 4	4 + 4 + 4	4 + 4 + 4
Vestibular kineties, number	ND	3	3–4	3–4
Postoral kineties, number	ND	6–7	5–7	7–8
Paroral membrane	ND	One-rowed, very close to vestibular kinety	One-rowed, very close to vestibular kinety	Double-rowed
Dorsal zone of parallel kineties	ND	8–10	6–8	Not applicable
Micronucleus, number	ND	3–9	1–3	Not observed
Swimming rotation	ND	R / L	R / L	L
Reference	[29, 30]	Present study	Present study	[15]

*ND* No data, *L* During swimming cell rotates to the left, *R / L* During swimming, cell can rotate in both directions – right (predominantly) and left, *PCV* Pore of contractile vacuole

According to some morphological characteristics, *F. vernalis* (neotype) recalls *F. shii,* but the two species cannot be synonymised. The number of somatic kineties (120–145 vs. 128–142), the number of vestibular (3 vs. 3–4) and postoral kineties (6–7 vs. 7–8) are similar between *F. vernalis* (neotype) and *F. shii* (Table 7)*.* However, *F. vernalis* (neotype) cells are smaller (250 × 125 μm vs.300 × 200 μm in living condition), the position of its PCV is dorsal (vs.ventro-lateral), postoral suture ends before the posterior end of the cell (vs. reaches the posterior end of the cell), and it facultatively swims rotating to the left or to the right with respect to the longitudinal body axis (i.e., rotation is clockwise and anticlockwise vs. anticlockwise rotation only). Moreover, according to description, the PM of *F. shii* is formed by a double-rowed structure (vs. single-rowed) although, looking at the images provided of the silver stained cells, this arrangement is not so obvious (see Fig. 2G in [15]). A set of very distinctive features is present in *F. vernalis* (neotype), such as the number and type of Mi and the presence of parallel kineties in the dorsal apical areas (vs. absence in *F. shii*).

According to morphology, *F. paravernalis* differs from *F. shii*, being smaller in size (178 × 95 μm vs.300 × 200 μm in living condition), showing a lower number of somatic kineties (98–116 vs. 128–142), and showing different features regarding the PCV position, the PM, the postoral suture, and the swimming rotation (Table 7). *Frontonia paravernalis* clearly differs from *F. vernalis* (neotype) as well, showing a different combination of traits, such as the lower number of Mi (two vs. six on average), dorsal parallel kineties (6–8 vs. 8–10), and the presence of a fourth row of VK (vs. only three in *F. vernalis* from present study).

The distance among the 18S rDNA sequences of these ciliates (*F. vernalis, F. paravernalis*, and *F. shii*) corroborates morphological investigation findings, further confirming their attribution to different species: on average their sequences diverge for ten nucleotides or more, that is a rather conspicuous difference for this highly conserved molecular marker. Indeed, what we suspect is that species belonging to the green frontoniids clade would constitute a complex of species. Obviously, further molecular studies would be required to address this issue.

Comparison between morphology of Italian and Russian populations of *F. paravernalis* indicates that morphometric values are very similar among the two populations (Table 4) (mean values): body size - 159.0 × 90.2 μm vs. 171.7 × 100.4 μm; somatic ciliature (ventral + dorsal) - 105 vs. 95; Ma size - 48.5 × 23.2 μm vs. 52.3 × 28.4 μm; Mi size - 3.9 × 2.5 μm vs. 3.4 × 2.6 μm. The main characters present quite stable features as well: i) in cells of both populations the number of the Mi range from one to three (two on average); ii) cells possess one CV with one PCV; iii) the pattern of the oral ciliature is identical, i.e., consists of peniculi with four rows of kinetidies each, single-rowed PM closely associated with the first VK; 3–4 VKs; 5–7 PKs; iv) longitudinal dorsal strip of kinetids as well as structure and position of CV are identical (Fig. 4g, Suppl. Fig. 1).

Interestingly, algal symbionts in the investigated populations of *F. paravernalis* are molecularly different between Italian and Russian populations (see Discussion below). The nature of Feulgen-positive particles observed in the cytoplasm of cells in Italian population of *F. paravernalis* (Fig. 4a, c-e) remains unknown. FISH results indicate that they are not *Eubacteria*, and TEM observations confirmed this point; thus, further investigations are needed to solve this interesting issue.

To sum up, according to the 18S rDNA-based phylogenetic reconstruction we can treat all green frontoniids as members of the same cluster and, in our opinion, they might possibly form a complex of species given that i) from a morphological point of view all of them manifested a single CV and the symbiosis with *Chlorella*-like algae - indeed, we believe that the capability to host green algae as cytoplasmic symbionts should be treated as a stable feature of green frontoniids as well; and ii) they all share the freshwater environment.

### The *Frontonia* type species issue

The type species of genus *Frontonia* has never been precisely designated and type material is presently lacking. As already mentioned, the first two species of *Frontonia* ever described were *F. vernalis* and *F. leucas* sensu Ehrenberg, firstly indicated as *Bursaria* [29] and then renamed after *Frontonia* [30].

As extensively discussed above, *F. vernalis* sensu Ehrenberg lacks a proper description and several subsequent misidentifications occurred since its first publication, suggesting that this species would not be the best candidate to became the type species of the genus.

On the other hand, neither original description of *F. leucas* sensu Ehrenberg, was exhaustive. Ehrenberg [29, 30] provided only few morphological details: *F. leucas* presented a single CV (“*die sternfӧrmige contractile Blasé*”) and green inclusions in the cytoplasm were absent [30]. Despite these inconveniences, in our opinion, *F. leucas* could be the best candidate as the type species of genus *Frontonia.*

Foissner and colleagues provided a detailed morphological redescription of *F. leucas* [3], but unfortunately, they did not provide a type material and molecular markers for the studied ciliate. Once more in that publication *F. leucas* was not designated as the type species of the genus *Frontonia*, although it was suggested later on by another author [45].

According to literature data [3, 4], *Frontonia leucas* morphospecies presents a wide morphological variation, but actually, it is more likely that different species of *Frontonia* have been often misidentified as *F. leucas*, given that in phylogenetic reconstructions these organisms are located far away to each other (see for example sequences MG437395–96, in Fig. 6).

Indeed, in a very recent article dealing with the description of *F. leucas* and three additional *Frontonia* species isolated in Turkey with the phylogenetic reconstruction of the genus, Kizildag and Yildiz wrote: “We guessed that the identification of this sequence [*F. leucas* – AM0722622, published in [9]] might be incorrect, since the morphological data were not presented” ([25]: p. 561). In our opinion, the above sentence is even logically incorrect: the lack of specific morphological data associated to *F. leucas* AM0722622 in the investigation by Fokin et al. [9] actually was only due to the necessities of the research context, i.e., the study was focused on the description of *Apofrontonia dohrni*.

However, with the present study, we took this opportunity to integrate the molecular data (i.e., the first sequence published under the name of *F. leucas*, AM072622) with the diagnosis (morphological description plus figures) of the *F. leucas* Italian population IPBG. Therefore, we propose this population of *F. leucas* corresponding to the sequence AM0722622 as the neotype of the species.

Moreover, concerning the identification of the organisms named after *F. leucas* (sequences MG437395–96) by Kizildag and Yildiz [25], we should express some doubts. Turkish populations of putative “*F. leucas”* ([25]: p. 560) significantly differ from the morphological descriptions of the “classical” *F. leucas* ([3]: pp. 169–170) in several important features (i.e., number of somatic kineties, number and size of the Mi, structure of CV) (details in Table 8). Indeed, according to the presented phylogenetic analysis, those organisms belong to the clade of *F. paramagna* [46], and, definitely, the strains described by Kizildag and Yildiz [25] are also more similar to *F. paramagna* ([46], present study: see Table 8) than to *F. leucas* morphotype. Actually, the only character that differs between “*F. leucas*” sensu Kizildag and Yildiz [25] and *F. paramagna* is the Mi (single and big vs. several and small). However, authors do not show in their paper any picture of “*F. leucas*” Mi, which can only be observed in provided drawings; therefore, it cannot be excluded that they might have overlooked the presence of several relatively small Mi.

**Table 8:** Comparative morphology of selected freshwater *Frontonia* species

Characters	***F. leucas*** sensu Foissner	***F. leucas*** Italy, Neotype	***F. leucas*** India	***F. leucas*** Turkey	***F. paramagna*** China	***F. paramagna*** India	***F. angusta*** Neotype	***F. angusta*** Turkey
Average body length (μm)	250	180	173	335	341	293	116	170
Cytostome/body length ratio	1/5–1/8	1/7	1/6	1/8–1/9	1/10	1/10	1/5	1/4
Somatic kineties, number	115	104	100	187	180–200	166–208	94	105
Peniculi (I, II, III), number	4–5, 4–5, 4–5	5, 5, 5	5, 5, 5	4–5, 4–5, 4–5	4, 4, 4	4, 4, 4	5–4, 3, 2	4–5, 4–5, 4–5
Vestibular kineties, number	3	3	3	3	3	3	4	3
Postoral kineties, number	6	5–6	6	6–8	6–7	6–7	4	4–6
Micronuclei, number	2–9	2–3	2–4	1	8^a^	6–14	1	1
Micronuclei, size (μm)	ND	3–5	1.5–2	7–8^a^	ND	1.5–2.3	7^a^	5–8
CVC, number	~ 10	8–11	7–8	10–12	~ 15	10–14	Absent	Absent
CVC, structure	Long, twisted	Long, twisted	Long, twisted	Branched	Long, straight	Long, straight	Absent	Absent
PCV, number	1	1	1	1	1	1–3	2–4	1
References	[3, 4]	Present study	Present study	[25]	[15, 46]	Present study	[7]	[25]

*CV* Contractile vacuole, *CVC* Canals of contractile vacuole, *ND* No Data, *PCV* Pores of contractile vacuole

^a^According to figures

As for the other *Frontonia* spp. studied by Kizildag and Yildiz [25], the ciliate corresponding to the sequence indicated as “*Frontonia angusta*” (MG456580) in our opinion could have been similarly misidentified: the sequence clusters with *F. leucas* (AM072622, KY855558), and the ciliate presents a morphology closer to that of our *F. leucas* from India (population KNP3), rather than *F. angusta* sensu Foissner et al. [3], which is the paper presenting neotypification. For further details see Table 8.

To summarize, since: i) the sequences presented by Kizildag and Yildiz [25] under the name “*Frontonia leucas*” (MG437395–6) cluster in the *F. paramagna*-clade (significantly far from the type sequence of *F. leucas*- AM072622); ii) the organisms the sequences (MG437395–6) belonged to are morphologically similar to *F. paramagna*; and iii) “*Frontonia angusta*” (MG456580) does not match the morphology of the neotype proposed by Foissner et al. [3], we strongly believe that all those organisms were misidentified. They should be synonymised with *F. paramagna* and *F. leucas* respectively and renamed accordingly.

### *Frontonia* phylogeny

Our phylogenetical analysis is in line with the findings of previous studies on *Frontonia* and Peniculia [9, 11, 13–16, 25–28, 47–49]. As a first consideration, the monophyly of *Frontonia* was not supported by the 18S rDNA sequence-based phylogeny [11, 16, 26, 27, 46, 50]. Indeed, Clade 3 included the genus *Apofrontonia* and resulted to be sister of *Paramecium*-clade, while Clade 4 resulted to be sister to *Marituja-Stokesia-Disematostoma-*clade.

Analyzing morphological and ecological data from previous studies (for a review see [50]) some unifying traits for the four retrieved phylogenetic clades of *Frontonia* can be highlighted.

Clade 1 comprises medium-sized species (body length: ~ 100–300 μm in vivo) from brackish or marine habitats, generally showing a single CV, a number of somatic kineties comprised between 48 and 215, a single Mi (except *F. salmastra* showing 2–3 Mi), two to four ciliary rows in peniculus III, and a double-rowed PM.

Clade 2 includes medium/large-sized species (body length: ~ 170–600 μm in vivo) from freshwater habitats, generally showing a single CV (except *F. vesiculosa* showing several), a number of somatic kineties comprised between 92 and 208, multiple Mi, four ciliary rows in peniculus III.

Clade 3 comprises small-sized species (body length: ~ 70–150 μm in vivo) from brackish habitats, generally showing two CVs (except *F. didieri* showing only one CV), a number of somatic kineties comprised between 61 and 107, a single Mi, two to four ciliary rows in peniculus III.

Clade 4 comprises small-sized species (body length: ~ 60–120 μm in vivo) from freshwater and soil (*F. terricola*) habitats, generally showing one CV, a number of somatic kineties comprised between 40 and 90, one to two Mi, three ciliary rows in peniculus III.

It has to be mentioned that the analyzed traits, in some cases, are slightly variable depending on species and can overlap among groups (Clades 1–4), therefore we should be careful to use them as key characters for species identification or clade assignation, without a proper molecular characterization. Indeed, respect to the previous review made by Zhao and colleagues [50], some of such features seem to be rather unreliable, i.e., number of ciliary rows (2–4) in peniculus III for Clade 1 and Clade 3.

As a last consideration, being *F. leucas* the type species of the genus (herein formally established and neotypified), from a phylogenetic point of view, the “true” *Frontonia* species should be only those belonging to Clade 1 and Clade 2, which form a monophyletic group comprising the type species of the genus.

Therefore, the “*Frontonia*” species in Clades 3 and 4, in our opinion, should be attributed to two, or more, newly established genera. Thus, we strongly recommend such a revision for future studies on this topic.

### Endosymbionts of *Frontonia vernalis* and *F. paravernalis* sp. nov.

The phylogenetic analysis of *Chlorella*-clade showed that *Chlorella* is a not monophyletic genus and phylogenetic relationships are not always resolved, confirming previous study results [51–53]. However, it is evident that endosymbionts of green *Frontonia* from Russian and Italian populations cluster significantly far away from each other, indicating an independent acquisition, affected by a sort of site-effect. Indeed, from molecular analysis, it came out that the type of green endosymbionts in the studied *Frontonia* populations differs depending on the site, rather than depending on the species: *F. paravernalis* from Serchio harbors the almost identical endosymbiont of *F. vernalis* from the same Italian site, but a different one with respect to *F. paravernalis* from St. Petersburg.

Additionally, *P. chlorelligerum* from the same location in St. Petersburg district, manifested very similar cytoplasmic alga phylogenetically close to *M. planctonica* [41]. Thus, it might be the case that, in old Peterhof water body, *M. planctonica* replaced *Chlorella* sp. as potential symbiont for different ciliates*.* Unfortunately, molecular investigation for algal symbionts of *F. shii* was not performed [15], therefore a comparison is unfeasible.

### Guideline proposal for an accurate morphological description of *Frontonia* species

With the present work, we would like to provide some useful and, in our opinion, substantial guidelines for the proper description of frontoniids based on literature data as well as on our own experience in the field. First of all, concerning the set of features to be considered for species discrimination, we want to stress several points: i) In case of frontoniids, usually, postoral suture is treated as a midventral line running from the oral area to the posterior pole of the ciliate; indeed, there could be a folding (a kind of comb of the cortex), which runs in parallel to a cortex line without kineties. So, the postoral suture is a combination of these two elements (Fig. 3). For instance, the preoral suture is always a line without kineties (Fig. 3). In other words, it is necessary to be precise whenever talking about “suture”, remaining strictly adherent to the exact morphological definition of the term. Errors in interpreting the proper morphological meaning of the postoral suture in *Frontonia* spp. can be found in some recent publications (see [28]: p. 314, Figs 1B, H and, in contrary, 1F as well as p. 317, Fig 3D, E; p. 319, Figs 5D, G and in contrary, Fig. 5E, F), where in different figures the same structure is presented either as a cortical comb or as an empty space between left and right parts of ventral side of frontoniid’s cell. ii) The characteristics of Mi (size, number, and type) should be considered as required features to be provided for any ciliate description. For Mi type identification see previous study on *Paramecium* [54]. iii) It is necessary to pay attention to the CV-associated structures: vesicle or canal type, number and structure of canals and PCV, location of CV. Especially for the genus *Frontonia*, it is important to record shape and interconnection of CV canals (e.g., are the channels straight, twisted, or forming a network – i.e., creating anostomosis among each other?). In some cases, the collecting canals are not easy to observe; the researcher should very carefully study several cells, not overly squeezing the object. iv) The trichocysts should be described according to their aspect in longitudinal view along with either the shape of their cross-section or their aspect viewed from the top. Indeed, aside from the more common circular cross section found in the extrusomes of the majority of *Frontonia* species, some trichocysts are more rhombic e.g., in *F. salmastra* [16], *F. marina* (Fokin, personal observations). Then, another point to be stressed is to use the important, but sometimes neglected, precaution to avoid providing contradictory data during species description. Unfortunately, it is a quite common error occurring in literature. For example, in a recent publication Cai and colleagues [15] propose a table ([15]: “Table 2”, p. 110) to summarise the main morphological characteristics for freshwater and soil frontoniids. Unfortunately, that table presents several inconsistencies: some of them could be treated as technical errors (e.g., the same number indicated body length and somatic kineties for *F. leucas* and *F. atra*; instead of number of CVs, the number of PCV is reported as 5–10 for *F. vesiculosa*), while some others show deeper misleadings (for details see: [17]), such as *F. pallida* [24, 55] and *F. elliptica* [18, 22], indicated as freshwater, instead of brackish water species. Moreover, according to Cai and colleagues [15] a soil species such as *F. terricola,* which lives in highly mineralized solution (in soil) was compared to freshwater species. Since it should be considered much more similar to brackish water species, it might have been avoided a comparison with freshwater ones.

## Conclusions

After a careful and critical revision of literature and according to our research data, we propose to reject *F. vernalis* sensu Ehrenberg [29, 30] as a valid description and we provide the neotypification of the species, based on the newly retrieved green frontoniid population from Italy (IPSal+b).

Moreover, in the present study we multidisciplinarly described a novel species of green frontoniid, i.e., *F. paravernalis* sp. nov., and we performed a critical revision of *Frontonia* phylogeny and literature, with special attention to *F. vernalis* and *F. leucas*. Some issues were fixed and the foundations for future studies that will further improve the state of the art on this genus were laid.

At present, what seems to be solid is that the green *Frontonia* representatives form a monophyletic clade and that all the more recently described species, i.e., *F. vernalis* (present study), *F. paravernalis* sp. nov. (present study), and *F. shii* [15], form a cluster of closely related freshwater ciliates with a single CV and hosting *Chlorella*-like organisms.

Concerning the critical discussion about the current status of *Frontonia* systematics, our contribution consisted in providing the 18S rDNA sequences of both, *F. vernalis* (neotype), *F. paravernalis* sp. nov., and of 14 other frontoniids isolated in different parts of the world over a research period of 15 years. Among them, at least two resulted new species (populations VmFr/IPSal- and BJ4), and four of them were already known frontoniids (*F. atra, F. fusca*, *F. minuta,* and *F. vesiculosa*) for which the gene sequence was obtained for the very first time.

The last contribution of our work dealt with the neotypification of *F. leucas* through the deposition of the neotype material in a museum collection, and the formal establishment of *F. leucas* as type species of the genus which was heretofore lacking. Filling this gap, in our view, was definitely crucial in order to properly plan further studies on *Frontonia* and the revision of its systematics.

## Methods

### Sampling sites and conditions

Green *Frontonia* spp*.* were repeatedly sampled in Italy between May 2016 and June 2018 (in details: May and November 2016, February and March of 2017, June 2018) in the permanent freshwater shallow small pond located along the Ligurian sea coastline close to the mouth of Serchio River (Parco Naturale di Migliarino San Rossore Massaciuccoli, Migliarino, Pisa district, Tuscany, Italy; N. 43° 47′ 7.524″ E. 10° 15′ 57.44″): populations IPSal+sm, IPSal+b (Table 1). Water temperature in sampling site ranged between 14 and 25 °C; pH was 7.6–7.8.

The Russian populations of green *Frontonia* spp. were collected in the period 2014–2017 during all seasons in Old Peterhof, in the small, but relatively deep permanent ditch in the corner of the English park: Peterhof, St. Petersburg district, Russia (N. 59° 52′ 45.88′′ E. 29° 51′ 37.224′′): populations FSPBsm, FSPBb (Table 1). Water temperature in sampling site ranged from 1 °C (January) up to 22 °C (August). The pH was 6.6–7.1.

Two different kinds of green *Frontonia* spp. were observed in both the samplings (i.e., in Italy and in Russia) based on the cell size: the “small” cell (IPSal+sm, FSPBsm) and the “big” cell (IPSal+b, FSPBb) populations. After a deep investigation, two different species were recognized and decribed: *F. paravernalis* sp. nov. (IPSal+sm, FSPBsm) and *F. vernalis.* (IPSal+b). The “big” cell population FSPBb was identified as *F. vernalis* on morphological base; due to the lack of molecular data on this population, it is not possible to assign it with certainty (see Results).

Additional *Frontonia* spp. were collected during our sampling activity carried out from 2005 to 2017 in Italy (Tuscany, Sardinia) and in India (Andhra Pradesh), as listed in Table 1. Some of the retrieved populations were maintained as polyclonal cultures for some time in the laboratory. Several of them, were recognised as *F. atra*, *F. minuta, F. vesiculosa, F. paramagna, F. leucas*, and *F. fusca* [10], described according to morphological and molecular criteria, and herein used for phylogenetic reconstruction of the genus *Frontonia*. In the case of *F. fusca*, the article with the redescription of the ciliate was published already a decade ago [10].

### Living cultures

Unfortunately, we did not succeed to establish monoclonal cultures of green frontoniids, but they could be kept in laboratory for some weeks/months (depending on the population) by feeding them with *Phaeodactylum tricornutum* (diatom, monoclonal culture) and *Peridinium* sp. (dinoflagellate, native isolate), in the original Falcon tube (50 ml) used for sampling. Specimens of green *Frontonia* spp. collected by micropipette could survive inside 3 ml-depression slides for some weeks, without feeding, but with day-time illumination.

### Live observations

Ciliates were observed using differential interference contrast (DIC) microscopy with a Leitz (Germany) microscope at a magnification of × 300–1250 with the help of a compression device [56]. Ciliates were photographed using a digital camera (Canon Power Shot S45) and True Chrome HDII Screen. Morphometric measurements were made both in vivo and after staining (i.e., after Feulgen staining and silver impregnation).

For the examination of swimming behaviour, ciliates were observed in a glass 3 ml-depression slide under a dissection microscope Wild M3 (Switzerland) at a magnification of × 12.5–50. Photoreactivity of ciliates was roughly checked in small Petri dishes, half decorated by dark case and illuminated by natural or artificial light.

### Fixation, staining and fluorescence in situ hybridization

Investigated ciliates were fixed with Champy’s solution and silver nitrate stained after Corliss [57]. Feulgen staining procedure after fixation in Bouin’s fluid or in a mixture of 95% alcohol with 1% solution of celoidin in diethyl ether (our own recipe) was used to reveal the nuclear apparatus. In order to check the possibility that the Feulgen-positive particles observed in the cytoplasm of IPSal+sm population were symbiotic bacteria, ciliates were processed for Fluorescence In Situ Hybridization (FISH) using the *Eubacterial* probe EUB338I [58] and the *Alphaprotobacterial* probes Alf1b [59]. Fixed cells were observed under UV-light with fluorescent microscope Leica DMR (Germany).

### Transmission electron microscopy (TEM)

TEM was used to investigate the nuclear apparatus, possible intracellular symbionts aside of algae and other cytoplasmatic structures in *F. paravernalis* sp. nov.. Cells were processed through a routinely used protocol [60].

### DNA extraction

The *Frontonia* cells were washed several times in glass depression slides with sterile distilled water before being fixed in 70% ethanol. Total genomic DNA was extracted from 40 (IPSal+sm), 30 (IPSal+b) and 20 (IPSal-) cells using the NucleoSpinTM Plant II kit (Macherey-Nagel, Germany).

### Whole genome amplification

The single cell whole genome amplification was performed on green *Frontonia* populations with symbiotic associations with *Chlorella*-like alga (IPSal+sm and IPSal+b from Italy; FSPBsm and FSPBb from Russia), using REPLI-g kit (Qiagen, Hilden, Germany). Cells were prepared with a series of six washing steps in distilled water inside glass depressions using glass micropipette, and finally cells were transferred to PBS. Then, a single cell of *Frontonia* plus 4 μL of PBS was withdrawn from the depressions and transferred to PCR microtubes. Next steps were performed according to manufacturer instruction. A microtube with only PBS and reagents from the kit was used as negative control.

### 18S rDNA amplification and sequencing

Polymerase chain reactions (PCR) on the 18S rDNA gene, were performed with a C1000TM Thermal Cycler (Bio-Rad, Hercules, CA) employing TaKaRa PCR reagents and Ex *Taq* (Takara Bio Inc., Japan). All PCRs were performed in a 40 μl reaction volume following the manufacturer’s instructions.

The amplification of 18S rDNA sequences from IPSal+sm, IPSal+b, and IPSal- populations was performed using DNA material from total genomic extraction, while amplification for FSPBsm and FSPBb populations was performed starting from DNA material obtained from WGA.

For PCR on IPSal+sm and IPSal+b we employed the forward primer 18S F9 (5′- CTG GTT GAT CCT GCC AG -3′) [61] and the reverse primer 18S R1513 Hypo (5′TGA TCC TTC YGC AGG TTC -3′) [62, 63] with the following settings: 180″ at 94 °C, followed by a series of 35 identical amplification cycles (denaturation at 94 °C for 30″, annealing at 55 °C for 30″, extension at 72 °C for 120″). The amplification of IPSal- was done on a diluted extraction product using forward primer 18S F9 Euk and reverse primer R1513 Hypo with the following settings: 180″ at 94 °C, followed by a series of 45 identical amplification cycles (denaturation at 94 °C for 30″, annealing at 55 °C for 30″, extension at 72 °C for 120″). A seminested PCR was performed on the PCR product for IPSal- using forward primer 18S F9 and Penic R1280 (5′- CGA CAC GTC CTA ACA AGA-3′) for the first reaction and using forward primer Penic F82 (5′-GAA ACT GCG AAT GGC TC-3′) and reverse primer 18S R1513 Hypo for the second reaction. The amplification of the samples was obtained using the following settings: 30″ at 94 °C followed by a series of 40 identical cycles (denaturation at 94 °C for 30″, annealing at 48 °C for 30″, extension at 72 °C for 120″).

To obtain the 18S rDNA sequence from FSPBsm and FSPBb populations we performed a seminested PCR starting from the WGA product with primers 18S F9/Penic R1280 and Penic F82/18S R1513 Hypo, as described for IPSal- population. PCR products were purified using EuroGOLD Cycle-Pure Kit (EuroClone, Milan, Italy) and sent to GATC Biotech Company (Germany) where the samplese were sequenced using three internal primers.

#### *Frontonia 18S rDNA* sequencing

For populations IPSal+sm and IPSal+b we employed the following sequencing primers: 18S R536 (5′-CTG GAA TTA CCG CGG CTG-3′), 18S R1052 (5′-AAC TAA GAA CGG CCA TGC A-3′), and 18S F783 (5′-GAC GAT CAG ATA CCG TC-3′) [64].

For IPSal- population we employed Penic R661 (5′- ACT AAT GCC CCC ATC TGT-3′), Penic R1280, and Penic F987 (5′-GGT CAA AAC ATG GAT GGG A-3′) as sequencing primers.

For populations FSPBsm and FSPBb we used 18S Penic R661, 18S R1052, and 18S F783 as sequencing primers. Unfortunately, we did not get suitable results for *Frontonia* FSPBb from Russia.

As for the other *Frontonia* in analysis (Table 1), they were processed for 18S rDNA amplification and sequencing according to Serra et al. [65].

#### *Endosymbiont 18S rDNA* sequencing

For the sequencing of the 18S rDNA of FSPBsm *F. paravernalis’* endosymbionts we employed the primers 18S R536 and 18S R1052, and we designed the species-specific primer F838_Meyer (5′- GGA TGT TTC TTC GAT GAC TC-3′).

We faced several problems in sequencing the 18S rDNA segment of endosymbionts from populations IPSal+sm and IPSal+b using Sanger sequencing. Therefore, the DNA material obtained from WGA was processed with a Nextera XT library and sequenced at Admera Health (South Plainfield, USA), using Illumina HiSeq X technology to generate 75,709,674 and 74,962,776 reads (paired-ends 2 × 150 bp) for IPSal+sm and IPSal+b populations, respectively. Preliminary assembly of resulting reads from both the organisms was performed using SPAdes software (v 3.6.0) [66]. The complete 18S rDNA sequence belonging to the endosymbiotic *Chlorella*-like organisms, was computationally predicted from the assembled sequences using Barrnap [67], and manually verified via BLAST analysis.

### Phylogenetic analyses

The 18S rDNA of the studied *Frontonia* spp. and the algal symbionts were aligned with the automatic aligner of the ARB software package version 5.5 [68] on the SSU ref. NR102 SILVA database [69].

For the analysis of frontoniids, 71 sequences of other Oligohymenophoreans, 62 of which belonging to the subclass Peniculia (ingroup), were selected (dataset 1).

For the analysis on the symbionts, 46 sequences of other members of *Chlorella*-clade were selected, plus 7 other sequences belonging to *Parachlorella*-clade [51] as outgroup (dataset 2).

After manual editing to optimize base pairing in the predicted rRNA stem regions in each dataset, the two alignments were trimmed at both ends up to the length of the shortest sequence. A positional filter was applied to dataset 2, to keep only those columns where the most conserved base was present in at least 5% of the sequences. Resulting matrices contained respectively 1615 (dataset 1) and 1839 (dataset 2) nucleotide columns, which were used for phylogeny and for the identity matrix calculation.

For each phylogenetic dataset, the optimal substitution model was selected with jModelTest 2.1 [70] according to the Akaike Information Criterion. Maximum likelihood (ML) trees were calculated with the PHYML software version 2.4 [71] from the ARB package, performing 100 pseudo-replicates. Bayesian inference (BI) trees were inferred with MrBayes 3.2 [72], using three runs each with one cold and three heated Monte Carlo Markov chains, with a burn-in of 25%, iterating for 1,000,000 generations.

## Supplementary Information

**Additional file 1: Supplementary Figure 1.** Morphology of *Frontonia paravernalis* sp. nov. (Russian population – FSPBsm). a) Feulgen stained cell, showing nuclear apparatus: macronucleus (Ma) and micronuclei (Mi); b) ventral side of a silver stained specimen, showing oral and somatic ciliature. *Chlorella*-like endosymbionts (Ch) are visible behind the cortex as well; c) detail of oral aperture (OA). OA – oral aperture; P1, P2, P3 - first, second, third peniculus; PCV – pore of contractile vacuole; PK – postoral kineties; PM – paroral membrane; PtS – postoral suture; VK – vestibular kineties. Bars stand for 20 μm.

**Additional file 2: Supplementary Figure 2.** Morphology of *Frontonia leucas* (Italian population – IPBG). a) Living cell showing oral aperture (OA) and macronucleus (Ma) position; b) living cell showing trichocysts (Tc) under the cell cortex and contractile vacuole (CV) with its collecting canals (CC); c) detail of Ma and three micronuclei (Mi) in a living cell; d) Feulgen stained cell, showing nuclear apparatus: Ma and three Mi; e), f) ventral and dorsal somatic ciliature of a silver stained specimen; g) silver stained dividing cell; h) closer view of *F. leucas* oral ciliature after silver staining. CC – collecting canals; CV – contractile vacuole; Ma – macronucleus; Mi – micronucleus; OA – oral aperture; P1, P2, P3 - first, second, third peniculus; PCV – pore of contractile vacuole; PK – postoral kineties; PM – paroral membrane; PrS – preoral suture; PtS – postoral suture; Tc – trichocysts; VK – vestibular kineties; *Arrow* – dorsal set of kineties parallel to the preoral suture; *Double Arrowhead* – longitudinal dorsal stripe of adjacent kineties. Bars stand for 20 μm (a, b, e-h), 10 μm (c, d).

## Data Availability

ll data generated or analysed during this study are included in this published article. 18S rDNA sequences have been deposited in GenBank and accession numbers are showed in the Results section, in Table 1, in Figs. 6 and 7 of the present manuscript. Permanent slides are available from the corresponding author on reasonable request.

## Abbreviations

BI: Bayesian inference
CV: Contractile vacuole
FISH: Fluorescence in situ hybridization
Ma: Macronucleus
Mi: Micronucleus
ML: Maximum likelihood
PCR: Polymerase chain reactions
PCV: Pore of contractile vacuole
PK: Postoral kineties
PM: Paroral membrane
VK: Vestibular kineties
