# Supplementary figures and images for: The neotypification of Frontonia vernalis (Ehrenberg, 1833) Ehrenberg, 1838 and the description of Frontonia paravernalis sp. nov. trigger a critical revision of frontoniid systematics
Source: BMC Zool. 2021 Mar 8;6:4. doi: 10.1186/s40850-021-00067-9 (PMC10127333; doi:10.1186/s40850-021-00067-9)

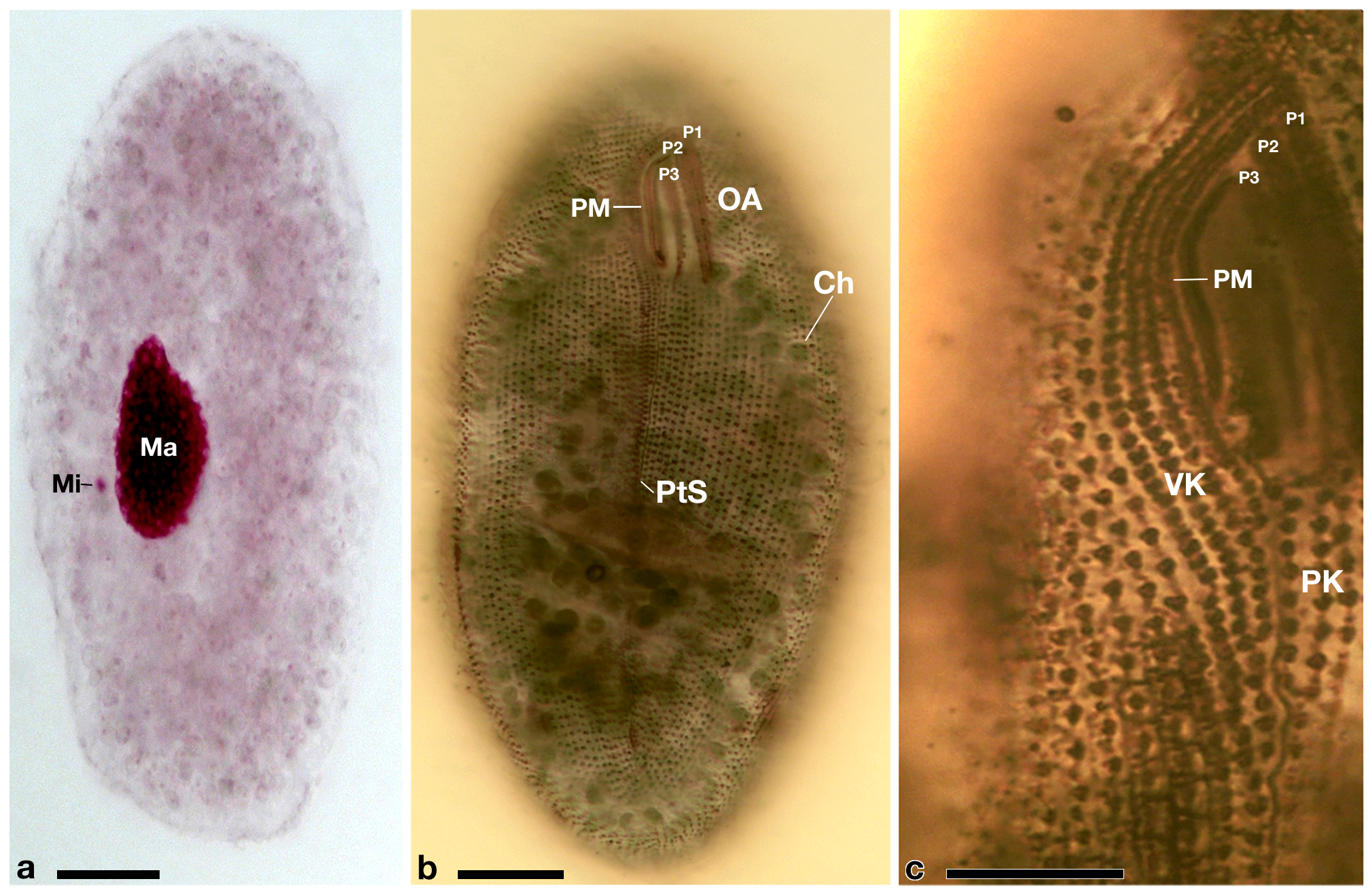

Supplement: Supplementary file 1 — Additional file 1: Supplementary Figure 1. Morphology of Frontonia paravernalis sp. nov. (Russian population – FSPBsm). a) Feulgen stained cell, showing nuclear apparatus: macronucleus (Ma) and micronuclei (Mi); b) ventral side of a silver stained specimen, showing oral and somatic ciliature. Chlorella-like endosymbionts (Ch) are visible behind the cortex as well; c) detail of oral aperture (OA). OA – oral aperture; P1, P2, P3 - first, second, third peniculus; PCV – pore of contractile vacuole; PK – postoral kineties; PM – paroral membrane; PtS – postoral suture; VK – vestibular kineties. Bars stand for 20 μm. [file 40850_2021_67_MOESM1_ESM.tif]

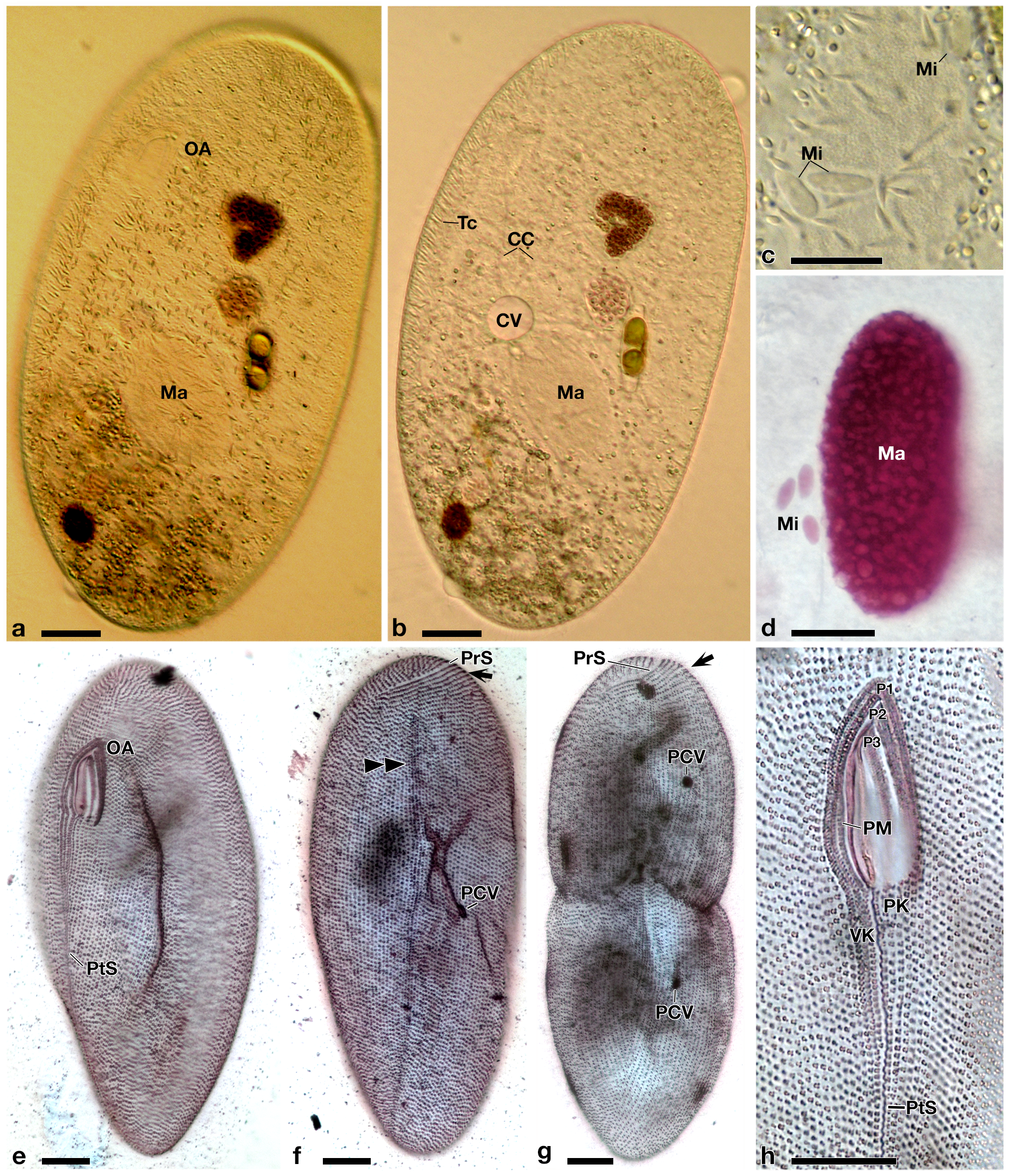

Supplement: Supplementary file 2 — Additional file 2: Supplementary Figure 2. Morphology of Frontonia leucas (Italian population – IPBG). a) Living cell showing oral aperture (OA) and macronucleus (Ma) position; b) living cell showing trichocysts (Tc) under the cell cortex and contractile vacuole (CV) with its collecting canals (CC); c) detail of Ma and three micronuclei (Mi) in a living cell; d) Feulgen stained cell, showing nuclear apparatus: Ma and three Mi; e), f) ventral and dorsal somatic ciliature of a silver stained specimen; g) silver stained dividing cell; h) closer view of F. leucas oral ciliature after silver staining. CC – collecting canals; CV – contractile vacuole; Ma – macronucleus; Mi – micronucleus; OA – oral aperture; P1, P2, P3 - first, second, third peniculus; PCV – pore of contractile vacuole; PK – postoral kineties; PM – paroral membrane; PrS – preoral suture; PtS – postoral suture; Tc – trichocysts; VK – vestibular kineties; Arrow – dorsal set of kineties parallel to the preoral suture; Double Arrowhead – longitudinal dorsal stripe of adjacent kineties. Bars stand for 20 μm (a, b, e-h), 10 μm (c, d). [file 40850_2021_67_MOESM2_ESM.tif]
